# Understanding *Bartonella*-Associated Infective Endocarditis: Examining Heart Valve and Vegetation Appearance and the Role of Neutrophilic Leukocytes

**DOI:** 10.3390/cells13010043

**Published:** 2023-12-25

**Authors:** Kristians Meidrops, Valerija Groma, Niks Ricards Goldins, Lauma Apine, Sandra Skuja, Simons Svirskis, Dita Gudra, Davids Fridmanis, Peteris Stradins

**Affiliations:** 1 Riga Stradins University, 16 Dzirciema Street, LV-1007 Riga, Latvialaumaapine.e@gmail.com (L.A.); peteris.stradins@stradini.lv (P.S.); 2 Centre of Cardiac Surgery, Pauls Stradins Clinical University Hospital, 13 Pilsonu Street, LV-1002 Riga, Latvia; 3 Joint Laboratory of Electron Microscopy, Riga Stradins University, 9 Kronvalda Boulevard, LV-1010 Riga, Latvia; 4 Institute of Microbiology and Virology, Riga Stradins University, Ratsupites Str. 5, LV-1067 Riga, Latvia; ssvirskis@latnet.lv; 5 Latvian Biomedical Research and Study Centre, LV-1067 Riga, Latvia; dita.gudra@biomed.lu.lv (D.G.); davids@biomed.lu.lv (D.F.)

**Keywords:** infective endocarditis, cardiac valves, vegetation, neutrophils, neutrophil extracellular traps, *Bartonella* spp.

## Abstract

Background. The endocardium and cardiac valves undergo severe impact during infective endocarditis (IE), and the formation of vegetation places IE patients at a heightened risk of embolic complications and mortality. The relevant literature indicates that 50% of IE cases exhibit structurally normal cardiac valves, with no preceding history of heart valve disease. Gram-positive cocci emerge as the predominant causative microorganisms in IE, while Gram-negative *Bartonella* spp., persisting in the endothelium, follow pathogenic pathways distinct from those of typical IE-causing agents. Employing clinical as well as advanced microbiological and molecular assays facilitated the identification of causative pathogens, and various morphological methods were applied to evaluate heart valve damage, shedding light on the role of neutrophilic leukocytes in host defense. In this research, the immunohistochemical analysis of neutrophilic leukocyte activation markers such as myeloperoxidase, neutrophil elastase, calprotectin, and histone H3, was performed. A distinct difference in the expression patterns of these markers was observed when comparing *Bartonella* spp.-caused and non-*Bartonella* spp.-caused IE. The markers exhibited significantly higher expression in non-*Bartonella* spp.-caused IE compared to *Bartonella* spp.-caused IE, and they were more prevalent in vegetation than in the valvular leaflets. Notably, the expression of these markers in all IE cases significantly differed from that in control samples. Furthermore, we advocated the use of 16S rRNA Next-Generation Sequencing on excised heart valves as an effective diagnostic tool for IE, particularly in cases where blood cultures yielded negative results. The compelling results achieved in this study regarding the enigmatic nature of *Bartonella* spp. IE’s pathophysiology contribute significantly to our understanding of the peculiarities of inflammation and immune responses.

## 1. Introduction

Infective endocarditis (IE) is a severe infectious disease that affects the heart’s endocardium and cardiac valves. The incidence of IE has increased two-fold over the last two decades [1], with the annual incidence being around 3–19/100,000 of the population [1,2]. Despite the use of effective antimicrobial therapy, the mortality rate of IE patients remains unacceptably high—up to 30% in 30 days to 1 year [2,3]. The most common complications of IE are congestive heart failure, paravalvular abscess formation, and embolism [4]. Vegetation growth identifies IE patients at high risk of embolic complications and death [1,5,6]. Large aortic valve vegetations are associated with a higher incidence of surgery and abscess formation in older patients with IE [7,8]. However, both the factors that influence the size of vegetation, when first detected, and the prognostic implications of the size of the vegetation have not been clearly determined [7,9].

The known pathophysiology of IE is one example of how the immune system has a connection with the hemostatic system. The endothelial lining of the heart and its valves are normally resistant to infection with bacteria and fungi and maintain their functions [10,11]. Conversely, the formation of valve vegetation may be induced by a damaged or inflamed endothelium [12,13,14]. Congenital heart valve diseases like the bicuspid aortic valve and altered valve leaflets due to calcification are predisposing factors for IE endothelium damage and vegetation formation [3,6,12]. However, the pertinent literature suggests that 50% of IE cases demonstrate structurally normal cardiac valves and no prior heart valve disease [13].

In blood culture-positive IE (BCPE), Gram-positive cocci as causative microorganisms are commonly detected [14]. In high-income countries, *Staphylococcus aureus*, *Streptococcus* spp., and *Enterococcus* spp. are confirmed as causes of IE in 25–40%, 17, and 10% of cases, respectively [3,14]. In turn, the incidence of blood culture-negative IE (BCNE) varies geographically but remains high, up to 30–70% [15,16,17]. The presence of fastidious or slow-growing organisms, such as *Haemophilus* spp., *Aggregatibacter* spp., *Cardiobacterium hominis*, *Eikenella corrodens*, *Kingella* spp. (s. HACEK group organisms), *Coxiella* spp., and *Bartonella* spp., as well as various laboratory techniques for identifying bacteria in blood cultures, antibiotic use, and low detectable levels of bacterial growth, collectively contribute to BCNE. Gram-negative IE is rare. Up to 3–4% of endocarditis cases are caused by *Bartonella* spp., which is a Gram-negative, facultative intracellular parasite with a likelihood of pathogenic pathways different from those of common IE causative agents. Persisting in the endothelium, *Bartonella* is shown to demolish the architecture of the valve and enhance remodeling [18]. After the innate immune system recognizes the invader, *Bartonella* is eliminated from the bloodstream but is still present in the valvular endothelial cells (VECs), where microorganisms aggregate and continue spreading [18]. Intracellular signals of *Bartonella* spp. are also demonstrated to increase the proliferation of VECs. In this case, bacterial colonization of the vegetation is a complex process involving interaction between the infecting organism, VECs, valvular interstitial cells (VICs), blood platelets, and potential biofilm formation [18].

Opportunistic pathogenic microorganisms from skin lesions, dental infections, intravenous devices, and intravenous drug users’ injection sites invade the bloodstream, causing an immune system response through the release of cytokines. Neutrophils, as innate immune system first-line phagocytes, induce neutrophil extracellular trap (NET) formation through netosis [19]. NETs comprise DNA meshwork packed with highly destructive enzymes like myeloperoxidase (MPO), neutrophil elastase (NE), and calprotectin (CP) [20]. DNA-citrullinated histones H3 are shown to be particularly toxic to endothelial cells [21]. Due to the endothelium injury and increased procoagulant activity through released cytokines and exposed collagen, prothrombotic extracellular matrix components and thrombocyte adhesion to damaged cardiac leaflets are induced. The vegetation built by the NETs binding the von Willebrand factor (vWF) to endothelium and platelet interaction can initially be sterile, but due to pathogenic microorganisms in the bloodstream and the adhesive NET meshwork, microorganisms are gradually entrapped [13,22]. Neutrophils activated by thrombocytes continue netosis on the top of the leaflets. The immune system’s response to pathogens induces chemoattraction of cells, continues leaflet tissue remodeling, and induces neo-angiogenesis [23]. Over time, *Staphylococcus aureus* and *Streptococcus*, initially trapped in NETs, are shown to escape phagocytosis and elimination by inhibiting NETs [22,24]. Several studies have implicated the involvement of NETs in the development and progression of infectious diseases, including endocarditis caused by different pathogens [25,26]. However, the specific interactions between *Bartonella* spp. and NETs in the context of IE remain poorly understood.

In the current study, we looked into the intricate morphology of the changed heart valves connected to vegetation development in IE patients. We investigated this on various levels, using clinically relevant non-invasive vegetation visualization techniques, established microbiological and cutting-edge molecular biology assays for IE causative pathogen testing in excised valve tissues, and various morphological techniques for examining the damage a disease causes to the heart valves and the role of neutrophilic leukocytes in the presentation of host defenses. The heart valve tissues used as controls were obtained from patients undergoing cardiac surgery. Clinically, transthoracic (TTE) and transoesophageal (TEE) echocardiography for the overall detection of vegetation in IE patients was used. Conventional microbiological analyses along with 16S rRNA Next Generation Sequencing were used to detect a pathogen. Routine histopathology was applied to identify the valvular damage. Taking advantage of the variety of immunohistochemical staining used, we confirmed the presence of NET-constituting enzymes and neutrophilic leukocyte DNA-citrullinated histones H3 in the samples studied. Transmission electron microscopy, as a gold standard for studying changes at the cellular level, was used to explore the intimate interactions between the host and the invading pathogen. Special attention was devoted to the ultrastructural characteristics displayed by neutrophilic leukocytes. In turn, scanning electron microscopy was used to illustrate the damage caused to the valvular endothelium and explore a sandwiched pattern of vegetation architecture. An extensive bioinformatics approach was used to analyze the associations between the studied variables. Given that the pathophysiology of *Bartonella* IE is still poorly understood, understanding the interaction between *Bartonella* spp. and NETs is crucial for unraveling potential pathogenic mechanisms. We believe that some new insights into the contributions of host-derived immunologic responses and pathogen-associated virulence factors to morphologic traits came to light and were clarified in this study.

## 2. Materials and Methods

### 2.1. Patients’ Characteristics and General Clinical Data

The cohort of 46 adult IE patients with indications for cardiac surgery were included in the study. Patients were operated on at Pauls Stradins Clinical University Hospital, Riga, Latvia from June 2020 to August 2022. Endocarditis diagnosis was based on the Duke criteria established by cardiologists and cardiac surgeons. Indications for cardiac surgery were based on the 2015 ESC European guidelines for the management of infective endocarditis. Conventional blood and cardiac valve microbiological cultures and 16S rRNA NGS testing were used to explore IE causative pathogens. To better explore the appearance of the heart valve and formation of vegetation in *Bartonella* spp.-associated IE, the subjects enrolled in this study were further stratified into three groups: (i) 10 patients with *Bartonella* spp.-caused IE, (ii) 12 patients with other pathogen-caused IE, and (iii) a control group of 23 patients. Other pathogens assessed were extracellular microorganisms, Gram-positive pathogens such as *S. aureus*, *E. faecalis*, and *Streptococcus* spp., and coagulase-negative Staphylococci. To better understand the extent of neutrophilic activation, a control group for morphological investigation was created and analyzed. Controls were patients who underwent elective cardiac surgery and received aortic or mitral valve replacement due to significant valvular insufficiency without IE. These patients were specially matched to ones in the IE groups with the same valvular type (aortic or mitral), gender, and age. In the present study, antibiotic therapies were regimented to the recommendations of the 2015 ESC European guidelines for the management of infective endocarditis and were based on the causative microorganism confirmed and antibacterial susceptibility. The study was approved by the Central Medical Ethics Committee of Latvia (Decision No. 5070, 22 May 2020) and conducted according to the Declaration of Helsinki. Informed consent was obtained from all study participants. Other medical data, such as patients’ anamnesis, laboratory analyses, and clinical imaging data, were obtained from medical records.

### 2.2. Methods of Microbiological Investigation

The IE patients’ blood was collected from a vein using the closed blood collection system needles, holder, and BacT/ALERT media. Negative results for aerobic and facultatively anaerobic bacteria were issued after 5 days, and for anaerobic bacteria after 7 days. Samples from the positive culture bottles were stained with Gram and examined under a microscope. MALDI-TOFF Sepsityper identification and preliminary sensitivity to antibiotics by disc diffusion test were determined. Confirmatory culturing on Columbia blood agar, McConkey plate, mannitol-salt agar and, if necessary, also on chocolate agar or Sabouraud medium, was performed. MALDI-TOFF technology was used in the identification of grown colonies. Antimicrobial susceptibility was determined by the disk diffusion method as well as the VITEK2 and broth microdilution methods. The results were interpreted based on the current version of the EUCAST recommendations. The valve tissue specimens obtained at the time of surgery were transported in a sterile container. To prevent it from drying out, physiologic saline solution was added to the sample. In the laboratory, the sample was cultured on Columbia broth, Columbia blood agar, MacConkey agar, mannitol-salt agar, and Sabouraud agar at 36 ± 1 °C for 24 h. A MALDI TOF mass spectrometer was used for colony identification. Besides conventional microbiological analyses, a small piece of the excised valvular vegetation was placed in a sterile specimen collection container (Sarstedt AG & Co. KG, Nümbrecht, Germany), immediately frozen to −20 °C, and transported to a laboratory for further processing. Altogether, 46 samples from 46 patients were obtained and analyzed.

#### 2.2.1. Microbial DNA Extraction for 16S rRNA NGS Analyses

Microbial DNA from the cardiac valve tissue was extracted using the FastDNA Spin Kit for Soil (MP Biomedicals, Irvine, CA, USA), according to the manufacturer’s guidelines. The concentration of extracted DNA was measured using a Qubit 2.0 Fluorometer and Qubit dsDNA HS Assay Kit (Life Technologies, Waltam, MA, USA). Altogether, 46 samples from 46 patients were obtained and analyzed.

#### 2.2.2. 16S rRNA Gene V3-V4 Amplification and Illumina MiSeq Sequencing

The two-stage PCR protocol was applied for MiSeq library preparation. Primers were designed for PCR amplification of the 16S rRNA gene V3-V4 region specific to the domain bacteria (341F and 805R, respectively) [27] containing Illumina overhang adapters (Table 1). Microbial DNA (4 ng) was amplified separately by V3 and V4 primers using Phusion U Multiplex PCR Master Mix (Thermo Fisher Scientific Waltam, MA, USA) under the following reaction conditions: denaturation at 98 °C for 30 s, 35 cycles of 98 °C for 10 s, 67 °C for 15 s, 72 °C for 15 s, and fragment elongation at 72 °C for 7 min. The yield of the acquired PCR products was assessed using 1.2% agarose gel electrophoresis. These were further purified using a NucleoMag NGS Clean-Up and Size Select kit (Macherey-Nagel, Duren, Germany). The concentration of the PCR products was measured using a Qubit dsDNA HS Assay Kit and a Qubit 2.0 Fluorometer, and samples were normalized to 4 ng/µL. During the second PCR stage, Illumina MiSeq i7 and i5 indexes were added to 4 ng of V3 and V4 PCR product using custom-ordered Nextera XT Index Kit (Illumina Inc., San Diego, CA, USA) primers (Metabion International AG, Planegg/Steinkirchen, Germany). For this reaction, Phusion U Multiplex PCR Master Mix was used under the same thermal cycler reaction conditions as specified for the first PCR stage. The 16S rRNA PCR products were then pooled and purified for the sequencing reaction using NucleoMag magnetic beads. The quality and acquired amount of the 16S rRNA V3-V4 amplicons were assessed using an Agilent High Sensitivity DNA Chip kit, an Agilent 2100 BioAnalyzer (Agilent Technologies, Santa Clara, CA, USA), a Qubit dsDNA HS Assay Kit, and a Qubit 2.0 Fluorometer.

Prior to sequencing, all samples were pooled at equal molarities and diluted to 6 pM. They were then paired-end sequenced using a 500-cycle MiSeq Reagent Kit v2 and an Illumina MiSeq (Illumina Inc.). Each run was expected to produce at least 10,000 reads per sample. After the sequencing run was completed, the individual sequence reads were filtered using MiSeq Reporter Software v.2.6 (Illumina, San Diego, CA, USA) to remove low-quality sequences. All MiSeq quality-approved, trimmed, and filtered data were exported as fastq files.

#### 2.2.3. 16S Sequence Analysis

Sequence reads were demultiplexed using Illumina’s MiSeq Reporter Software v.2.6 (Illumina, San Diego, CA, USA) and quality filtered using Trimmomatic [28] v.0.39, with the leading quality of Q20 and trailing quality of Q20; sequences shorter than 36 nucleotides were discarded. All quality-approved sequences were imported into the QIIME 2 v.2021.11 environment for further analysis. The DADA2 [29] plug-in was used to pair forward and reverse reads, as well as for extra sequence quality control and chimeric sequence removal using a pooled consensus method. The resulting feature table and sequences were used for de novo clustering employing the VSEARCH plug-in using a 97% identity threshold [30]. Thereafter, de novo multiple sequence alignments were performed using the MAFFT method [31], while phylogenetic trees were constructed using FastTree 2 [32]. De novo clustered sequences were used for taxonomic assignment with the pre-fitted learn-based [33] taxonomy classifier based on the SILVA v.132 97% identity reference database, which was trained with the Naïve Bayes classifier.

### 2.3. Histopathological and Histochemical Investigation of the Heart Valve Alteration in IE

The specimens used in this study were obtained during cardiac surgery. A small piece of the excised heart leaflet with vegetation was further processed for molecular testing and for growing microorganisms through culturing. The remaining part of the sample, if it was sufficient and appropriate, was submitted for histopathological, histochemical, and immunohistochemical investigation. Altogether, suitable material for further morphological analyses was obtained from 22 patients with IE (10 *Bartonella* spp., 12 non-*Bartonella* spp.) and 23 controls. One out of the 11 *Bartonella* spp. patients’ valve leaflets and 16 out of the 28 patients’ valve leaflets were excluded due to inappropriate fixation or an insufficient amount of biological material. The valvular leaflets with macroscopically visible vegetation were fixed in 10% neutral formalin. Tissue samples were embedded in paraffin on the cut side from the free edge of the leaflet through the vegetation structure to the base of the leaflet. Conventional 4–5 µm thick tissue sections were cut off and mounted on SuperFrost Plus slides (Gerhard Menzel GmbH, Braunschweig, Germany). The sections were routinely stained with hematoxylin and eosin (H&E). Additionally, the reticulin silver plating kit, according to Gordon and Sweets (Merck KGaA, Darmstadt, Germany, 1002510001), was used to visualize reticular fibers within the leaflet tissue and, simultaneously, to better identify bacteria using the silver impregnation technique [34]. The presence of blood vessels within the leaflets was assessed in ten properly oriented microscopic fields for each region of interest and scored as follows: 0—absent; 1—fewer than five vascular profiles present; 2—five or more vascular profiles present. Similarly, the presence of neutrophilic leukocytes was estimated and scored as follows: 0—absent; 1—fewer than nine neutrophils present; 2—ten to forty-nine neutrophils present; 3—fifty or more neutrophils present.

### 2.4. Immunohistochemical Investigation of the Heart Valve Leaflets and Vegetations in IE

Formalin-fixed and paraffin-embedded (FFPE) samples were processed conventionally. Blocking of endogenous peroxidase activity was performed using 3% H_2_O_2_ in methanol. For specific antigen retrieval, sections were boiled in citrate buffer (pH 6) or TRIS/EDTA buffer (pH 9), as recommended by manufacturer protocols, and incubation with primary antibodies was conducted thereafter. For the recognition of various neutrophilic leukocyte activation markers, a broad spectrum of primary antibodies, including anti-myeloperoxidase (anti-MPO, Abcam, ab208670, 1:1000), anti-histone H3 (anti-Histone H3, Abcam, ab5103, 1:50), anti-calprotectin (anti-CP, Abcam, ab22506, 1:1000), anti-neutrophil elastase (anti-NE, Abcam, ab131260, 1:1000), and anti-P-selectin, CD62P (anti-Ps, Abcam, ab182135, 1:500), were used. Primary antibody amplification and visualization were performed using the HiDef Detection HRP Polymer system (No. 954D-30, Cell Marque, Rocklin, CA, USA) and the 3,3 diaminobenzidine (DAB) tetrahydrochloride kit (No. 957D-30, Cell Marque, Rocklin, CA, USA). Cell nuclei were counterstained with Mayer’s hematoxylin. Primary antibodies were omitted in the negative controls of immunohistochemical reactions. The reaction results were assessed by two independent observers. Microphotographs were obtained as captures using a Glissando Slide Scanner (Objective Imaging Ltd., Cambridge, UK). The expression of antigens in the valve leaflet and vegetation was assessed in ten properly oriented microscopic fields for each region of interest and scored as follows: 0—negative; 1—weakly positive; 2—strongly positive expression.

### 2.5. Transmission Electron Microscopy of the Heart Valve Leaflets and Vegetations in IE

Specimens, including the excised heart leaflet and vegetation, were collected during cardiac surgery and thereafter cut into 1 mm3 tissue blocks, and further processed for transmission electron microscopy (TEM). According to routine laboratory protocols, the samples were fixed in 2.5% glutaraldehyde in 0.1 M phosphate buffer, pH 7.2. The samples were postfixed in 1% osmium tetroxide, dehydrated, and embedded in epoxy resin (Carl Roth 8623.1, 8639.1). Ultrathin 60 nm thick sections were cut with an LKB ultramicrotome, collected on Formvar-coated 200-mesh nickel grids, and stained with 2% uranyl acetate and lead citrate. The sections were examined with a JEM 1011 transmission electron microscope (JEOL, Akishima, Tokyo, Japan) at a magnification range of × 6000 to × 50,000.

### 2.6. Scanning Electron Microscopy of the Heart Valve Leaflets and Vegetations in IE

For scanning electron microscopy (SEM) examination, the collected excised heart leaflet and vegetation samples were dehydrated in a series of graded solutions of acetone and dried in liquid CO_2_ using the critical point procedure (E3000 drying device, Laughton, UK) to preserve the specimen ultrastructure and provide a room temperature stable tissue. Thereafter, the specimens were mounted on a metal stub using a sticky carbon disc and silver-containing glue, coated with a gold layer, and examined by JSM-6490LV (JEOL, Akishima, Tokyo, Japan) with an accelerating voltage of 20 kV at a magnification range of ×500 to ×5000.

### 2.7. Statistical Data Analysis

The statistical analysis of data, along with graphing, was carried out using Prism 9 software for macOS (GraphPad Software, LLC, San Diego, CA, USA), JMP 16 (SAS, Cary, NC, USA), and Jamovi software v.2.4.8. The descriptive analysis encompassed calculating frequencies and percentages for categorical variables and determining the mean, median, standard deviation (SD), and interquartile range (IQR) for continuous variables. For inferential statistics, the variance of variables between patients’ positive and negative for *Bartonella* spp. was evaluated using multivariate Analysis of Variance (MANOVA). This analysis was conducted using the built-in stats package in the R environment. Comparison of intra- and extracellular expression of the potential biomarkers under study was analyzed using the Kruskal–Wallis test, followed by the two-stage linear step-up procedure of Benjamini, Krieger, and Yekutieli as the post hoc correction method. In cases where the Kruskal–Wallis test assumptions were not met, the Mann–Whitney U rank-sum test was used. To explore potential correlations between patients’ clinical and immunohistochemistry data, Spearman’s rank correlation analysis was employed. Correlation matrices and correlation clustering were employed to provide a clear overview of the associations between the intracellular and extracellular immunohistochemical variables under study. To minimize the impact of covariates as potential confounders on the corresponding pairwise associations, partial correlation analyses were conducted. Furthermore, to identify patterns within the collected data with respect to the studied individuals, a hierarchical clustering method was utilized to assess similarities and differences. Additionally, alluvial plots were generated to visually represent associations across categorical dimensions of variables. Statistical significance between variables was determined at a threshold of *p* < 0.05.

## 3. Results

### 3.1. Patients’ Characteristics, Laboratory Indices, and Clinical Outcomes in Non-Bartonella *spp.*- and Bartonella *spp.*-Caused IE

Among the 46 IE patients included in this study, 28 (60.9%) had positive blood cultures as confirmed with the use of conventional microbiological methods. There were 11 (23.9%) *Streptococcus* spp., 5 (10.9%) *S. aureus*, 5 (10.9%) *E. faecalis*, 4 (8.7%) other microorganisms, and 3 (6.5%) coagulase-negative staphylococci (CoNS) IE cases. There were 18 (39.1%) patients with no discovered causative agent of IE.

Overall, patients with *Bartonella* spp. had significantly lower platelet and leukocyte count, EF of the left ventricle, and glucose levels as well as higher BNP, and creatinine levels when compared to other microorganism-caused IE characteristics, laboratory indices, and clinical outcomes. Another finding was that patients with *Bartonella* spp. IE had a significantly higher rate of alcoholism history. Clinical outcomes, such as time spent intrahospital or in the ICU, as well as intrahospital death, were not significantly different. The IE patients’ characteristics observed in non-*Bartonella* spp. and *Bartonella* spp. groups are summarized in Table 2.

### 3.2. 16S rRNA NGS Testing Results for IE

All of the blood culture-positive IE pathogens were also detected by 16S rRNA NGS testing, with variable DNA relative abundance from 22.6 to 100%. A pathogen was detected in 12 (66.7%) of 18 BCNE cases using 16S rRNA NGS testing, with >90% relative abundance of bacterial nucleic acid. Unexpectedly, in 11 cases, it was *Bartonella* spp. infection. Of note, in all cases of *Bartonella* spp., the relative abundance of species detected by NGS was high and even reached 100%, except for two patients where it was 95.4 and 99.6%, suggesting this as the causative microorganism of IE. In one case, the *Streptococcus* spp. pathogen was found. Altogether, the detection of >90% relative abundance of bacterial nucleic acid was confirmed in 29 (63.0%) cases. Besides *Bartonella* spp.-caused IE cases, a relative abundance of 100% bacterial DNA was found in only 10.9% of patients in comparison to *Bartonella* spp. at 72.7%. The etiological agent was not found in six patients (13.0%), where blood and valve cultures were negative, and 16S rRNA NGS indicated the presence of multiple bacterial DNA, with one predominant microorganism’s relative abundance ranging from 31.5–71.7%, thus leaving the true causative agent unclear.

### 3.3. Histopathological and Histochemical Assessment of the Heart Valve Alteration and Vegetation in IE

**Figure 1:**
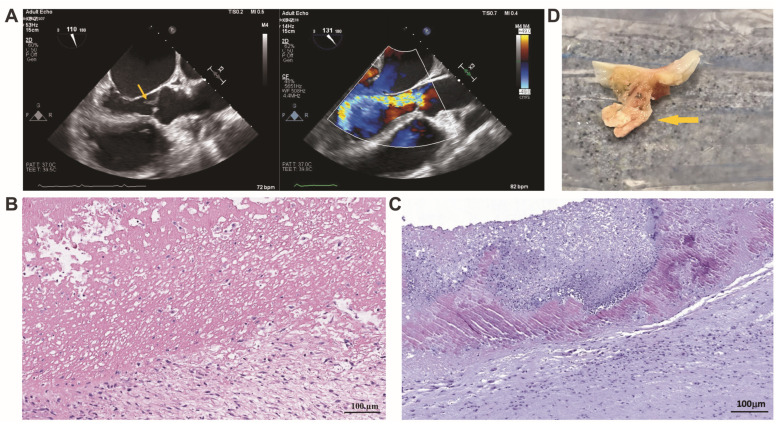
Transoesophageal echocardiography scan of *Bartonella* spp.-induced aortic valve endocarditis (**A**). The image on the left, marked with an arrow, depicts vegetation attached to the aortic valve leaflet, while the image on the right, using color Doppler, demonstrates severe aortic valve regurgitation. A representative image depicts the histopathological features of valvular vegetation visually attached to the leaflet in *Bartonella* spp.-caused IE. The cut-off region of vegetation reveals an amorphous material with a sponge-like appearance, which is invaded by immune cells. Fibroblasts and mononuclear cells are visualized in the leaflet fragment seen in this figure (**B**). Valvular vegetation in non-*Bartonella* spp. -caused IE with a bottom-side predominance of neutrophilic leukocytes, macrophages, and some bacteria interspersed by fibrin (**C**). H&E staining. Scale bar: 100 μm. Macroscopic appearance of excised aortic valve cusp with vegetation. The arrow indicates vegetation attached to the aortic valve leaflet (**D**).

Histopathologically, fibroblastic proliferation and a predominance of mononuclear cells were evidenced in the altered leaflets in *Bartonella* spp.-caused IE. Fibrotic changes and sometimes calcifications were detected as well. Sponge-like, eosinophilic vegetative masses were revealed in the specimens obtained from patients with *Bartonella* spp.-caused IE and confirmed with the use of imaging techniques (Figure 1A). A mass was composed of a fibrin meshwork and contained a low number of immune cells. A small number of microorganisms was present (Figure 1B). Similarly, eosinophilic vegetative masses were found in the specimens obtained from patients with non-*Bartonella* spp.-caused IE; however, the sponge-like vegetation mass was less accentuated than that appearing in *Bartonella* spp.-caused IE. Visually, a higher number of immune cells invaded a vegetative mass, and some microorganisms were present as well (Figure 1C).

**Figure 2:**
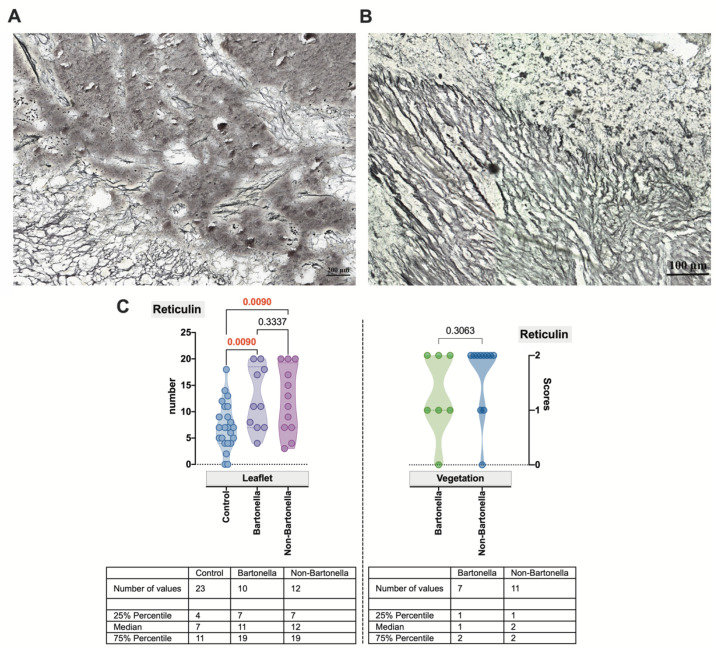
Representative image demonstrating clumps of bacteria found in vegetation in *Bartonella* spp. IE (**A**) and non-*Bartonella* spp. IE (**B**) samples. Silver stain demonstrates black coloration in clusters of *Bartonella bacilli*. A visible metallic tone produced by the silver solution highlights reticular fibers within the valve leaflet. Scale bar: 100 µm. Histochemically confirmed and scored data are plotted for controls, *Bartonella* spp., and non-*Bartonella* spp. within the valve leaflet (**C**: the plot on the left) and for *Bartonella* spp. and non-*Bartonella* spp. within vegetation (**C**: the plot on the right). Each dot represents a single data point. Abbreviations: Ctrl—control; Bartonella—*Bartonella* spp.-caused IE; non-Bartonella—non-*Bartonella* spp.-caused IE.

Thereafter, histochemical applications were employed to visualize the bulk of the cardiac valve connective tissue. To simultaneously label collagen reticular fibers and enhance the identification of bacteria, a reticulin silver plating kit was used. Within the leaflet, reticular fibers formed a network and enveloped an amorphous mass close to the vegetation in the specimens obtained from patients with *Bartonella* spp.-caused IE (Figure 2A). A slightly more parallel arrangement of reticular fibers was observed in non-*Bartonella* spp.-caused IE specimens (Figure 2B). This arrangement was found to have a statistically significant difference compared to that confirmed for control specimens’ cardiac leaflets (*p* = 0.0090) but not vegetations (Figure 2C). Clusters of black-colored bacilli were observed when silver impregnation was applied.

### 3.4. Immunohistochemical Assessment of the Expression of Markers of Neutrophil Activation Contributing to NETosis in the Heart Valve Leaflets and Vegetations in IE

To investigate the expression of various neutrophilic leukocyte granules, cytosolic enzymes, and histones in damaged cardiac valves and vegetations observed in both *Bartonella* spp.- and non-*Bartonella* spp.-caused IE, an immunohistochemical assay was used. Components of neutrophilic leukocyte azurophilic granules, including bactericidal agents such as NE and MPO, along with the cytosolic protein calprotectin and histone H3, were assessed both intra- and extracellularly. The valvular leaflets and vegetation were examined separately and compared to controls.

**Figure 3:**
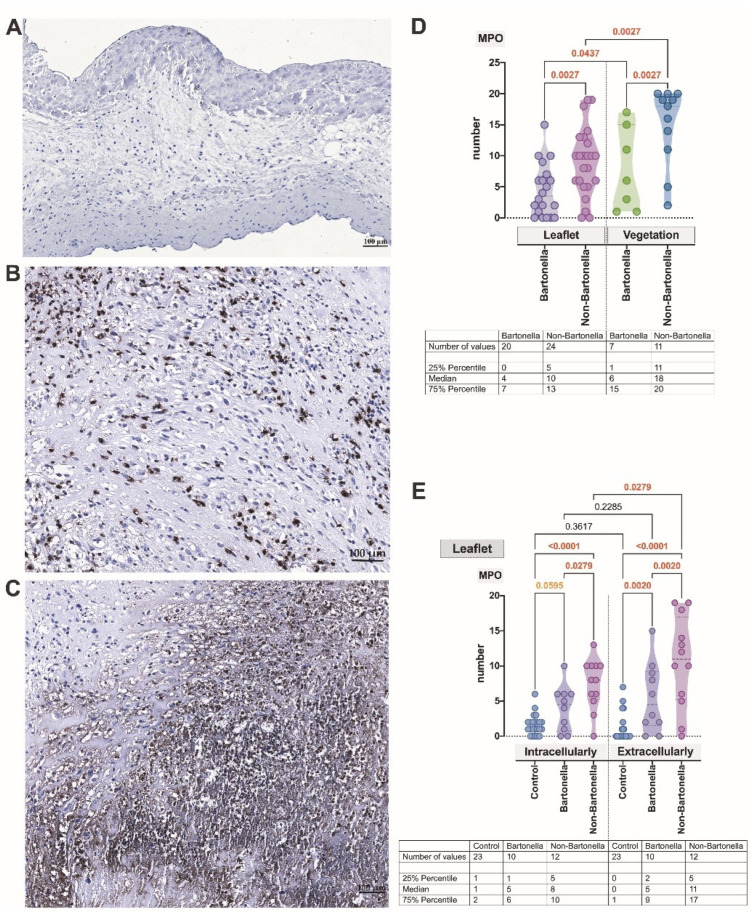
A representative illustration of immunohistochemistry reaction: negative control (**A**). Micrographs displaying the characteristic appearance of MPO expression observed intra- and extracellularly in the leaflet and valvular vegetation in *Bartonella* spp.-caused and non-*Bartonella* spp.-caused IE (**B**,**C**). The MPO statistics assessment (**D**,**E**). Abbreviations: MPO—myeloperoxidase; Bartonella—*Bartonella* spp.-caused IE; non-Bartonella—non-*Bartonella* spp.-caused IE. MPO immunohistochemistry. Scale bar: 100 μm.

A distinct difference was observed in the pattern of MPO distribution between *Bartonella* spp.-caused and non-*Bartonella* spp.-caused IE. The anti-MPO antibody exhibited strong staining of target cells; however, in non-*Bartonella* spp.-caused IE, it appeared more diffused and extended extracellularly when compared to *Bartonella* spp.-caused IE (Figure 3A–E). In *Bartonella* spp.- and non-*Bartonella* spp.-caused IE, some cells in the luminal space of the valvular neovessels were targeted. Notably, in vegetation, MPO expression was statistically stronger in both *Bartonella* spp.- and non-*Bartonella* spp.-caused IE (*p* = 0.0437 and *p* = 0.0027, respectively) (Figure 3D). In all IE cases, MPO expression significantly differed from controls, except intracellularly detected MPO in *Bartonella* spp.-caused IE. Both intracellularly and extracellularly, MPO was significantly more abundant in non-*Bartonella* spp.-caused IE when compared to *Bartonella* spp.-caused IE (*p* = 0.0279 and *p* = 0.0020, respectively) and controls (*p* < 0.0001). Simultaneously, in non-*Bartonella* spp.-caused IE, MPO was significantly more abundant (*p* = 0.0279) in the extracellular environment (Figure 3E).

**Figure 4:**
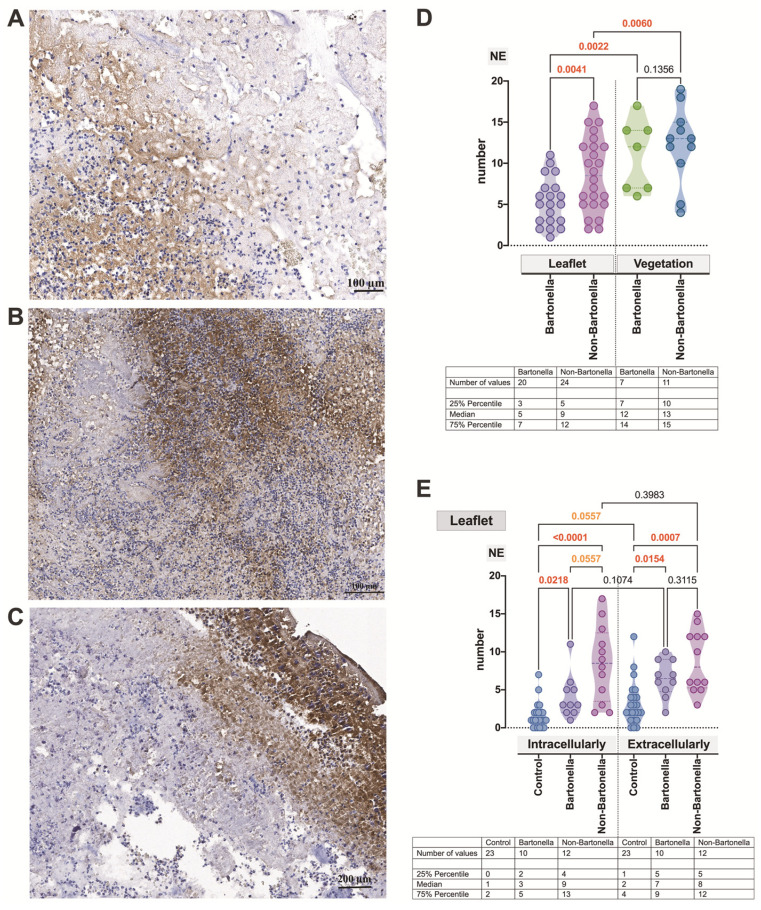
Micrographs displaying the characteristic appearance of NE expression observed intra- but mostly extracellularly in valvular vegetation in *Bartonella* spp.-caused IE (**A**) and leaflet and valvular vegetation in non-*Bartonella* spp.-caused IE (**B**,**C**). Vegetations are invaded by immune cells. The leaflets exhibit features of neovascularization. The NE statistics assessment (**D**,**E**). Abbreviations: NE—neutrophil elastase; Bartonella—*Bartonella* spp.-caused IE; non-Bartonella—non-*Bartonella* spp.-caused IE. NE immunohistochemistry. Scale bar: 100 μm.

The anti-NE antibody exhibited strong staining of target cells, accompanied by moderately strong and rather diffuse extracellular staining in both the leaflets and vegetations of both *Bartonella* spp.-caused and non-*Bartonella* spp.-caused IE (Figure 4A–C). Regarding the statistics assessment of this neutrophilic leukocyte activation marker, again, it was significantly stronger in vegetations compared to the leaflets (*p* = 0.0060 and *p* = 0.0022, respectively) and significantly prevailed in cardiac leaflets (*p* = 0.0041) in non-*Bartonella* spp.-caused IE (Figure 4D). Simultaneously, in both *Bartonella* spp.- and non-*Bartonella* spp.-caused IE, NE expression, whether intracellular or extracellular, was significantly higher (*p* = 0.0218, *p* < 0.0001 and *p* = 0.0154, *p* = 0.0007, respectively) compared to controls (Figure 4E).

**Figure 5:**
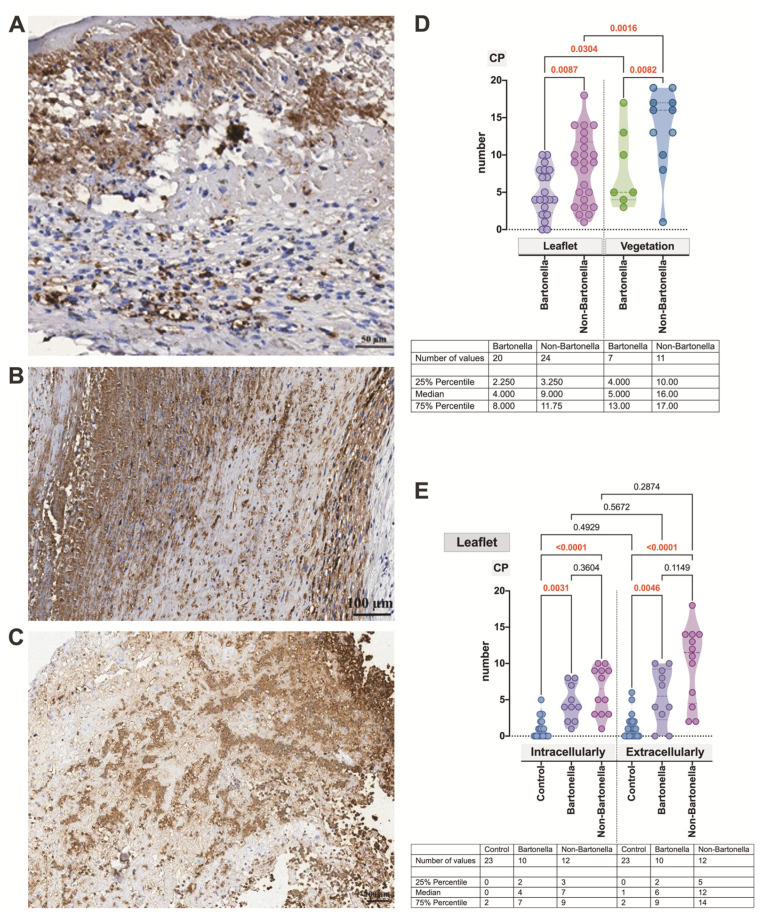
Micrographs displaying the characteristic appearance of CP expression observed intra- and extracellularly in valvular vegetation in *Bartonella* spp.-caused IE (**A**) and the leaflet and valvular vegetation in non-*Bartonella* spp.-caused IE (**B**,**C**). The leaflets exhibit features of neovascularization. The CP statistics assessment (**D**,**E**). Abbreviations: CP—calprotectin; Bartonella—*Bartonella* spp.-caused IE; non-Bartonella—non-*Bartonella* spp.-caused IE. CP immunohistochemistry. Scale bar: 100 μm.

As the next neutrophilic leukocyte activation marker, CP, an abundant cytosolic heterodimeric protein was investigated. The anti-CP antibody heavily decorated the cellular cytoplasm, while the extracellular staining pattern appeared more diffuse and smeared (Figure 5A–C,E). Similar to the previously mentioned activation markers, we observed a heightened expression of the marker in vegetation compared to the valvular leaflet (*p* = 0.0304 and *p* = 0.0016, respectively) for both *Bartonella* spp.-caused and non-*Bartonella* spp.-caused IE cases (Figure 5D). In both the leaflets and vegetation, the CP expression was significantly stronger in non-*Bartonella* spp.-caused IE cases (*p* = 0.0087 and *p* = 0.0082, respectively).

Finally, in both types of IE, CP expression, whether intracellular or extracellular, was significantly higher (Figure 5E) compared to controls (*p* = 0.0031, *p* < 0.0001 and *p* = 0.0046, *p* < 0.0001, respectively).

**Figure 6:**
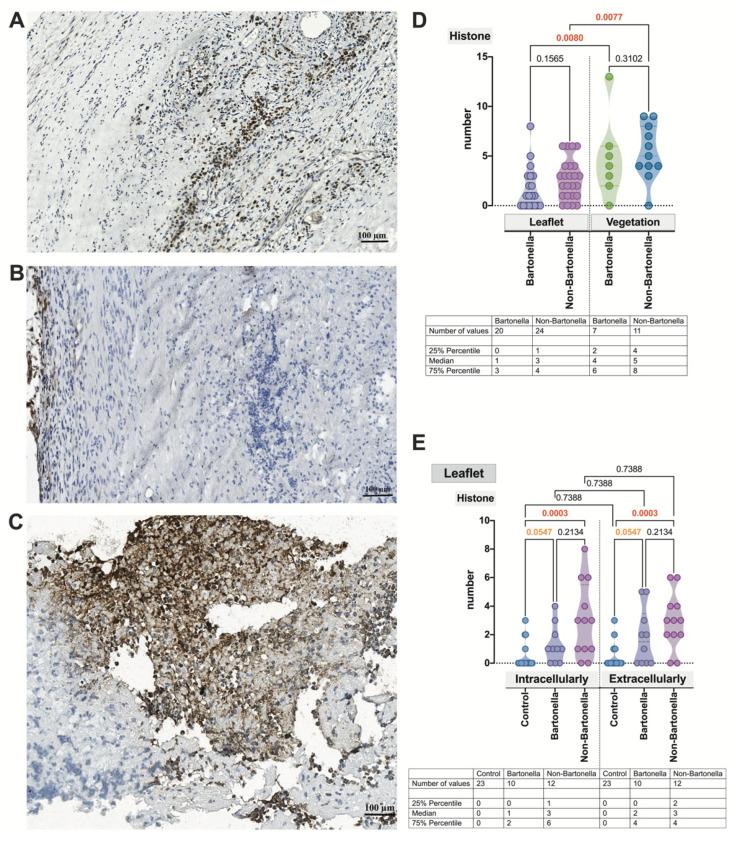
Micrographs displaying the characteristic appearance of histone H3 expression observed in the valvular leaflet in *Bartonella* spp.-caused IE (**A**) and the leaflet and valvular vegetation in non-*Bartonella* spp.-caused IE (**B,C**). The leaflets exhibit features of neovascularization. The histone H3 statistics assessment (**D**,**E**). Abbreviations: Histone—histone H3; Bartonella—*Bartonella* spp.-caused IE; non-Bartonella—non-*Bartonella* spp.-caused IE. Histone H3 immunohistochemistry. Scale bar: 100 μm.

The nuclear expression of the histone H3 marker was confirmed in all investigated cases of IE. In *Bartonella* spp.-caused IE (Figure 6A), the anti-histone H3 antibody clearly marked and outlined the cell nucleus, slightly overlapping the cytoplasm. Conversely, in samples obtained from non-*Bartonella* spp.-caused IE cases (Figure 6B), there was an appearance of an extracellular and smeared staining pattern. For both *Bartonella* spp.-caused and non-*Bartonella* spp.-caused IE cases, a significantly stronger expression of histone H3 was observed in vegetation compared to the valvular leaflet (Figure 6C,D) (*p* = 0.0080 and *p* = 0.0077, respectively). Importantly, histone H3 expression, whether intracellular or extracellular, was significantly higher (*p* = 0.0003) only in non-*Bartonella* spp.-caused IE cases compared to controls (Figure 6D).

**Figure 7:**
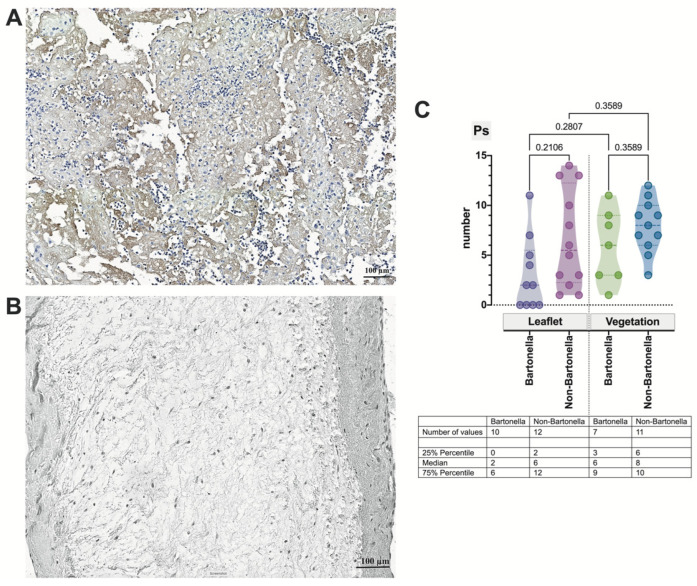
A representative illustration of Ps immunohistochemistry: vegetation attached to the damaged valvular leaflet in non-*Bartonella* spp.-caused IE (**A**) and the absence of reaction in the negative control (**B**). The micrograph demonstrates positive staining, likely reflecting the presence of this transmembrane protein in the blood platelets of vegetation (**B**,**C**). The region of interest is heavily colonized by neutrophilic leukocytes. The Ps statistics assessment (**C**). Abbreviations: Ps—P-selectin; Bartonella—*Bartonella* spp.-caused IE; non-Bartonella—non-*Bartonella* spp.-caused IE. Scale bar: 100 μm.

To further investigate the role of the adhesion molecule, P-selectin, in the formation of platelet-leukocyte conjugates, we applied P-selectin immunohistochemistry. While the damaged valvular leaflets showed some P-selectin expression, it was more pronounced in vegetation (Figure 7A,B). Notably, non-*Bartonella* spp.-caused IE cases exhibited a higher P-selectin expression compared to *Bartonella* spp.-caused IE (Figure 7C); however, the difference was not statistically significant.

### 3.5. Multivariate Analysis of Immunohistochemically Obtained Expressions of Neutrophilic Leukocyte Activation Markers

**Figure 8:**
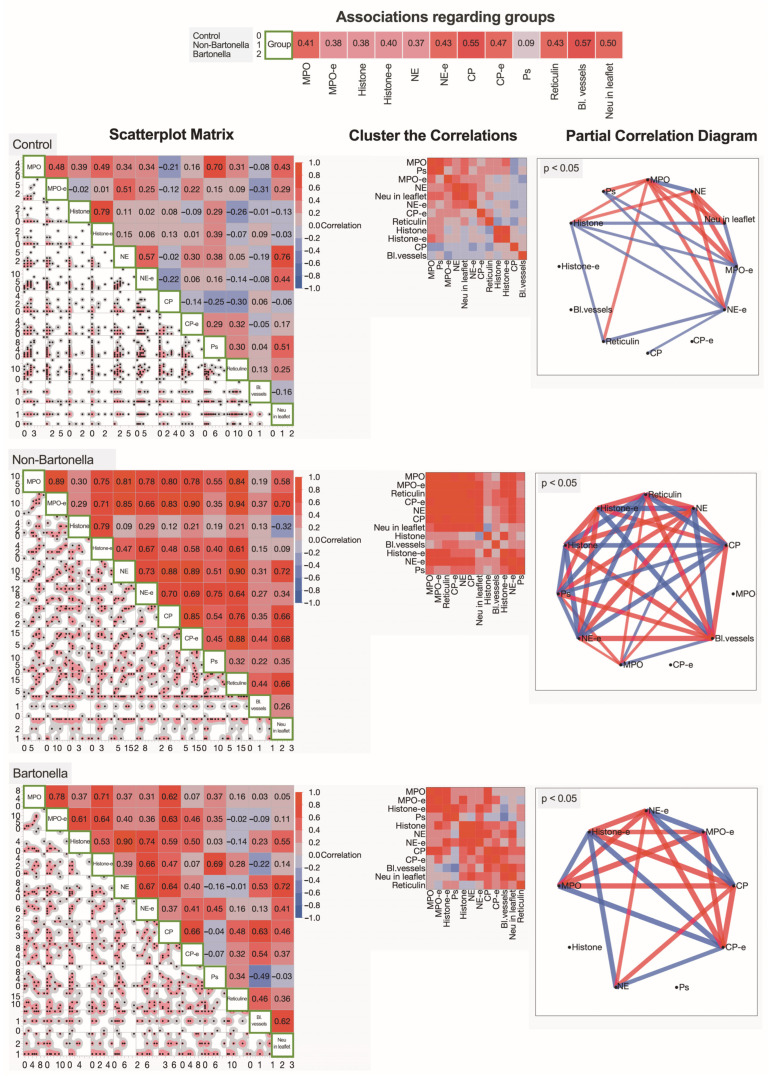
Associations between immunohistochemically obtained expressions of neutrophilic leukocyte activation markers. A scatterplot matrix, along with the corresponding Spearman’s rank correlation coefficients, correlation clusters, and partial correlation diagrams (on the **right**), is presented for the control group (**upper**), non-*Bartonella* spp. IE group (**middle**), and *Bartonella* spp. IE group (**lower**). Abbreviations: MPO—myeloperoxidase, MPO-e—myeloperoxidase expression extracellularly, Histone—histone H3 expression, Histone-e—histone H3 expression extracellularly, NE—neutrophil elastase expression, NE-e—neutrophil elastase expression extracellularly, Ps—P-selectin, Histone_v—histone H3 expression in vegetation, NE—neutrophil elastase, CP—calprotectin, CP-e—calprotectin expression extracellularly, Neu in leaflet—neutrophilic leukocyte count inside valvular leaflet, Bl.vessels—blood vessels inside valvular leaflet.

To represent the paired associations of expressed neutrophilic leukocyte activation markers, matrices of the Spearman’s rank correlation coefficients were generated for the control group and non-*Bartonella* spp. and *Bartonella* spp.-caused IE patient groups (Figure 8). For a more detailed overview of the interactions between the investigated neutrophilic leukocyte activation markers, the clusterization of correlations was presented for all three groups. Additionally, partial correlation diagrams illustrate the strength and direction of the most significant relationships between two variables, while controlling for the effects of covariates. All three groups of patients exhibited distinct neutrophilic leukocyte characteristics. In non-*Bartonella* spp.-caused IE patients, the content of the cytoplasmic granules of neutrophilic leukocytes appeared to strongly correlate with histones detected extracellularly, while in *Bartonella* spp.-caused IE patients, strong correlations of histones with NE and CP were detected intracellularly.

### 3.6. Ultrastructural Analysis of the Heart Valve Leaflets and Vegetations in IE

Employing transmission electron microscopy, which serves as the gold standard for investigating cellular-level changes, we delved into the close interactions between the host and the invading pathogen in the heart valve leaflets and vegetation in IE. Particular attention was focused on examining the ultrastructural characteristics exhibited by neutrophilic leukocytes during NET formation.

**Figure 9:**
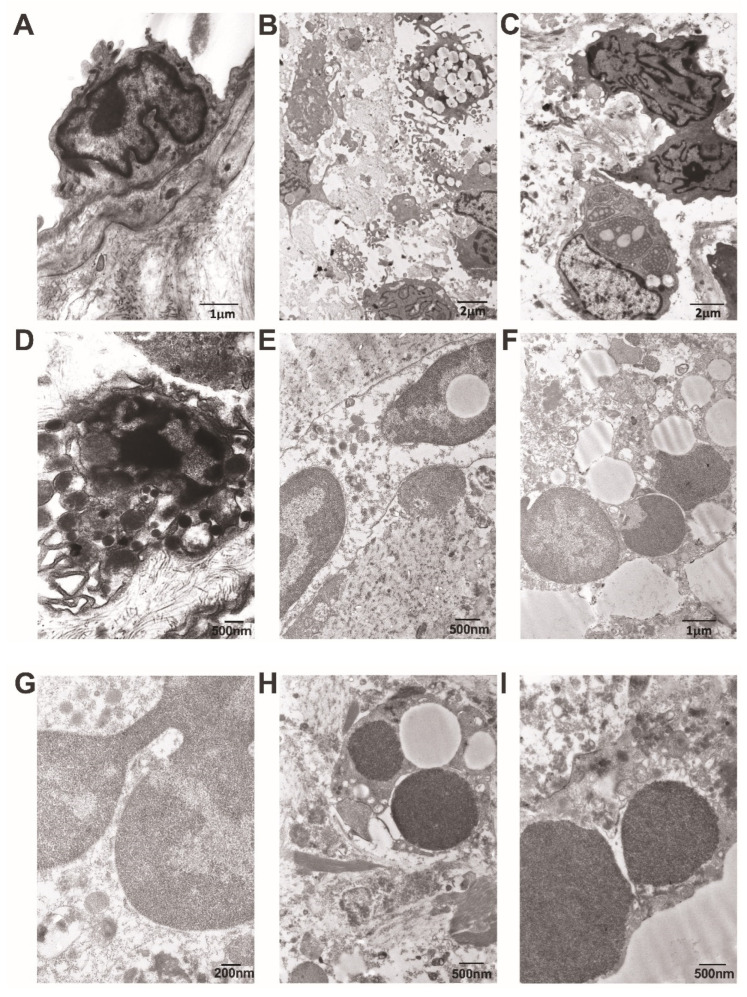
Representative images obtained using transmission electron microscopy. (**A**) The endothelium of the valvular leaflet in a *Bartonella* spp.-caused IE patient is roundly shaped, with few irregular and short microvilli, a wavy nuclear envelope, and marginated chromatin; cytoplasm reveals occasional organelles. (**B**) A low-power view of the damaged valvular leaflet in a non-*Bartonella* spp.-caused IE patient. Its content demonstrates various immune cells, including macrophages with well-developed pseudopodia and lipid-laden, are intermixed with collagen microfibrils. (**C**) In a region nearby, valvular cells reveal a very pronounced and deeply convoluted nuclear envelope; immune cells reveal the extensive expansion of the rough endoplasmic reticulum with content of varying density. (**D**) An irregularly shaped macrophage with a ruptured plasma membrane reveals a dilated perinuclear space, damaged mitochondria, and some cytoplasmic granules. (**E**) A neutrophil with a ruptured plasma membrane, wavy-outlined external nuclear membrane, and nuclear inclusion reveals a low-density hyaloplasm containing various granules. Clumps of neutrophil heterochromatin are positioned extracellularly. (**F**) The free apoptotic nuclei of a neutrophil are surrounded by extracellularly positioned clumps of heterochromatin, cellular debris, and lipid droplets. (**G**) A high-power view of the neutrophil reveals a nucleus with an irregularly dilated perinuclear space and a ruptured membrane. It is surrounded by a cytoplasm containing granules and autophagic vacuoles. (**H**) A fragment of the apoptotic neutrophil is enclosed by fibrin, collagen, and cellular debris. (**I**) A fragment of the neutrophil reveals an irregularly dilated perinuclear space with thread-like material, a ruptured membrane, and various granules positioned both intra- and extracellularly. Scale bars: 1 μm, 2 μm, 2 μm, 500 nm, 500 nm, 1 μm, 200 nm, 500 nm, and 500 nm.

Firstly, we examined the endothelial cells of the cardiac leaflet near vegetation (Figure 9A). These cells often appeared to have a rounded shape and displayed short and irregular microvilli. The contour of the nuclear envelope showed marked irregularity, and the nuclear chromatin exhibited moderate margination. The cytoplasm appeared electron-dense, revealing only a small number of organelles. Secondly, the observed vegetation consisted mainly of immune cells, primarily macrophages, plasma cells, and some platelets, which were sandwiched between fibrin threads (Figure 9B,C). In most cases, macrophages displayed well-developed, elongated pseudopodia used to engulf bacteria and cellular debris (Figure 9D). Finally, we paid special attention to the peculiarities of NET formation displayed by neutrophilic leukocytes (Figure 9E–I). The perinuclear space was often irregularly dilated, sometimes containing gentle material with a thread-like appearance. Chromatin, mostly condensed in our observations, frequently appeared extracellularly. Additionally, free apoptotic nuclei were visible. The cytoplasm exhibited low electron density and contained small, differently shaped granules. Some granules revealed a rupture of the membrane, and telolysosomes were evident as well. Mitochondria appeared to be small, and in some cases, they demonstrated a rupture of both the external and internal membranes. The plasma membrane often appeared ruptured, and cytoplasmic content and organelles were found in the extracellular space. Notably, in this investigation, the changes, characteristic of neutrophilic leukocytes, were more pronounced in cases of non-*Bartonella* spp.-caused IE.

**Figure 10:**
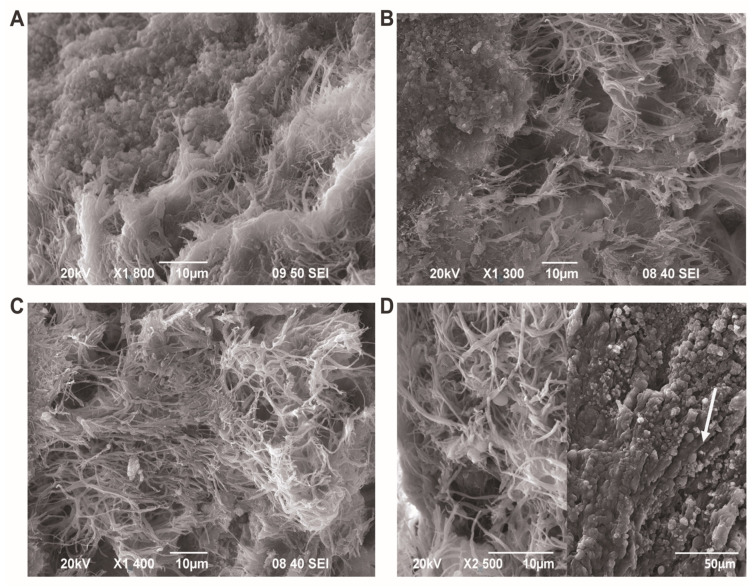
Representative scanning electron micrographs depicting the ultrastructure of the aortic valve endothelial cells and vegetation. (**A**) Low-power magnification showing the top and depth of vegetation. (**B**) Low-power depth view of vegetation with fibrin and a few platelets. (**C**,**D**) Vegetation side depth shows the sandwiched architecture of vegetation with a network of fibrin bundles and some bacteria trapped. When platelets, bacteria, and leukocytes form vegetation, the surface of tightly aligned endothelial cells (indicated by the white arrow) becomes barely visible. Scale bar: 10 μm; D panel on the right: 50 μm.

To enhance the visualization and illustration of bacterial adhesion to the valvular leaflet’s surface, the platelet appearance, and the formation of the fibrin network in IE caused by both *Bartonella* spp. and non-*Bartonella* spp. bacteria, as these appeared at the time of surgery, we used scanning electron microscopy. Our observations encompassed both the top and depth of vegetation, revealing bacteria attached to the endothelial surface. The well-developed organization of the fibrin network was evident, with some bacteria and blood cells visibly trapped (Figure 10 A–D).

### 3.7. Hierarchical Clustering Used for the Exploration of Data Similarities and Visualization Using Alluvial Plotting

**Figure 11:**
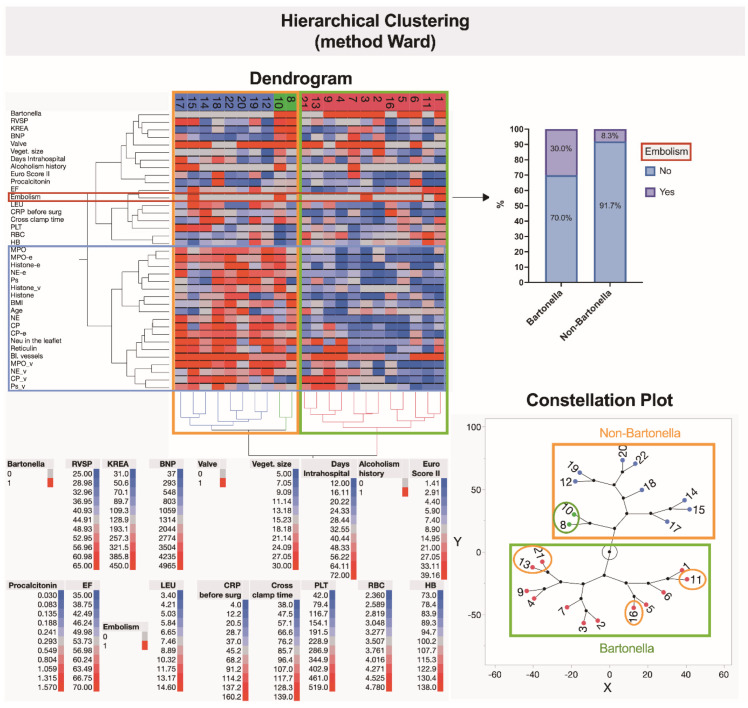
Hierarchical clustering shows the similarities of data points related to the studied system. The clades and leaves of a tree include data on the immunohistochemical evaluations of neutrophilic leukocyte activation markers, clinical parameters, complications, including and further specifying embolism, and several laboratory analyses. Variables are scaled in colors from blue to red, representing concentrations ranging from the lowest to the highest. Abbreviations: RVSP—right ventricle systolic pressure; KREA—serum creatinine level, BNP—B-type natriuretic peptide, Veget. size—vegetation size, Euro Score II—EuroScore II risk, EF—ejection fraction of the left ventricle, LEU—leukocyte count, CRP before surg.—C-reactive protein level before surgery, Cross clamp time—aortic cross clamp time, PLT—platelet level, RBC—red blood cell level, HB—hemoglobin level, MPO—myeloperoxidase, MPO-e—myeloperoxidase expression extracellularly, Histone-e—histone H3 expression extracellularly, NE-e—neutrophil elastase expression extracellularly, Ps—P-selectin, Histone_v—histone H3 expression in vegetation, BMI—body mass index, NE—neutrophil elastase, CP—calprotectin, CP-e—calprotectin expression extracellularly, Neu in the leaflet—neutrophilic leukocyte count inside valvular leaflet, Bl.vessels—blood vessels inside valvular leaflet, MPO_v—myeloperoxidase expression in vegetation, NE_v—neutrophil elastase expression in vegetation, CP_v—calprotectin expression in vegetation, Ps_v—P-selectin expression in vegetation.

This part of the study describes the application of a hierarchical clustering method to analyze similarities and differences in immunohistochemically obtained characteristics of neutrophilic leukocyte activation markers, clinical parameters, complications, and laboratory indices in cases of *Bartonella* spp.- and non-*Bartonella* spp.-caused IE. The hierarchical clustering analysis led to the recognition of three separate clusters of patients based on their characteristics and parameters (Figure 11). Interestingly, the analysis identified a noteworthy finding regarding the risk of embolism. The blue-colored cluster represents non-*Bartonella* spp.-caused IE patients exhibiting a notably high expression of neutrophilic leukocyte activation markers as assessed through immunohistochemical methods. Importantly, these patients did not show a significant risk of embolism. However, three of them had high leukocyte and platelet count values, and two had high right ventricle systolic pressure and C-reactive protein values and experienced issues related to alcoholism history. Two patients from a green-colored cluster had distinct characteristics that set them apart from the others. Analysis of a red-colored cluster presenting *Bartonella* spp.-caused IE patients revealed a notably high expression of neutrophilic leukocyte activation markers, including NE and CP. Additionally, high scores of neutrophils were observed in the valvular leaflets, although detected in fewer patients when compared to non-*Bartonella* spp.-caused IE cases. Importantly, two *Bartonella* spp.-caused IE patients presented with large vegetations, and two were noted to be at a high risk of embolism, suggesting that their condition might predispose them to this complication.

**Figure 12:**
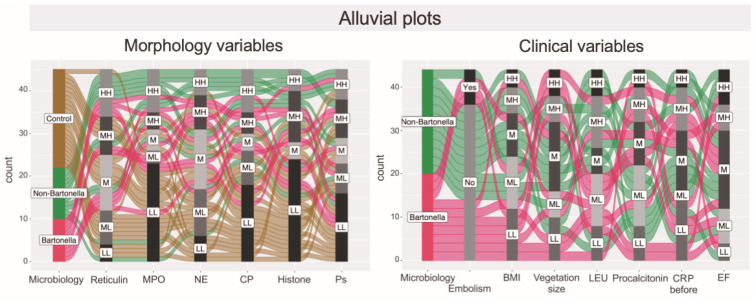
The alluvial diagrams represent flows among nodes. In the left diagram, individual assessments are presented as rows, while immunohistochemically obtained expressions of neutrophilic leukocyte activation markers are presented as columns. Statistically, the count for each marker is stratified into five different levels. In the groups studied, the width of each line depicted and the flow that stems from it are determined by the proportional fraction of the category total. The left plot clearly shows that in non-*Bartonella* spp.-caused IE, all immunohistochemical expressions indicative of neutrophilic leukocyte activation are mostly high and medium high. Simultaneously, the right plot shows that the levels of clinical parameters assessed in this study vary among the two groups: BMI, leukocyte count, procalcitonin, and CRO reached the highest level in non-*Bartonella* spp.-caused IE patients, while the largest vegetations were observed in *Bartonella* spp.-caused IE patients. Abbreviations: High, High (HH); Medium, High (MH); Medium (M); Medium, Low (ML); Low, Low (LL); MPO—myeloperoxidase; NE—neutrophil elastase; CP—calprotectin; Histone—histone H3; Ps—P-selectin; BMI—body mass index; LEU—leukocyte count; CRP before—C-reactive protein level before surgery; EF—ejection fraction of the left ventricle.

Furthermore, alluvial diagrams were plotted to illustrate the distribution of associations among categorical dimensions of variables. This form of data visualization was employed to enhance the clarity in depicting the distinct spectrum of variables linked to the immunohistochemically evaluated markers of neutrophilic leukocyte activation and clinical parameters in cases of *Bartonella* spp.-induced IE, non-*Bartonella* spp.-induced IE, and control subjects (Figure 12).

## 4. Discussion

The present study combines a diverse range of analytical methods and approaches to offer a deeper understanding of IE, particularly in the context of *Bartonella* spp.-caused cases, which are still relatively poorly understood. This study takes a multifaceted approach by analyzing a range of factors related to IE, including patients’ characteristics, laboratory indices, clinical outcomes, and histopathological assessments. It further delves into the immunohistochemical expression of neutrophilic leukocyte activation markers and markers associated with NETosis, providing a comprehensive view of the disease. Analysis of ultrastructural changes in IE further contributes to the advancement of knowledge in this field.

Microorganisms can evade phagocytosis, establish biofilms, and encapsulate themselves, resulting in their inaccessibility. While it has been widely held that cardiac vegetation can impede host defenses and prevent the clearance of bacteria, there remains an incomplete demonstration of the mechanisms underlying this phenomenon. According to McCormick’s (2002) observations, the characteristic lesion consists of bacteria enclosed within a layer of platelets and fibrin that adheres to the underlying endothelium [35]. Other researchers have indicated that these adhesive interactions persist during the subsequent transendothelial migration of leukocytes [36,37]. These interactions involve Mac-1, LFA-1, and their corresponding receptors ICAM-1 or members of the junctional adhesion molecule (JAM) family, alongside platelet endothelial cell adhesion molecule-1 (PECAM-1) or CD99-mediated homophilic interactions, as documented by Chavakis et al. in 2003 and Shaw et al. in 2004 [36,37]. Furthermore, it has been acknowledged that the infiltration of leukocytes to the site of infection necessitates their passage through the subendothelial extracellular matrix (ECM), which is guided by integrin-dependent adhesive interactions with matrix proteins. These proteins include fibrinogen, fibrin, fibronectin, and collagen, as highlighted by Triantafyllos Chavakis and colleagues in 2007 [38]. Importantly, the presence of mineralization has been linked to chronic cardiac valve infection in humans [39], as well as streptococcal IE in pigs [40]. Simultaneously, the diagnosis and pathophysiology of culture-negative *Bartonella* spp. endocarditis poses a considerable challenge, compounded by its high mortality rates. The intricate nature of this infection demands specialized assays for accurate confirmation. Few studies exemplify the successful diagnosis of such an elusive condition through a multifaceted approach, involving epidemiologic data, histological examination, and nucleic acid amplification testing [41]. In the present research, we delved into the complex structure of altered cardiac valves linked to the growth of vegetation in patients with IE. Our investigation covered multiple dimensions, employing pertinent non-invasive methods to visualize vegetation, well-established microbiological techniques, and advanced molecular biology assays to test for pathogens causing IE within excised valve tissues. We employed various morphological approaches to scrutinize the extent of valve damage induced by the disease and to elucidate the involvement of neutrophilic leukocytes in the presentation of the host’s defense response.

The histopathological examination of heart valve alterations and vegetations in IE provides insights into the distinct characteristics of *Bartonella* spp.-caused and non-*Bartonella* spp.-caused cases. Our study’s detailed immunohistochemical analysis of neutrophilic leukocyte markers such as MPO, NE, CP, and histone H3 highlights the differences in expression patterns between *Bartonella* spp.-caused and non-*Bartonella* spp.-caused IE. This sheds light on potential variations in the immune response and NETosis processes. Numerous proteins, including nuclear histones and cytoplasmic primary and secondary granule components, bind to NETs. Some of these components, such as NE, MPO, cathepsin G, and lactoferrin, exhibit bactericidal activity capable of eliminating microbial factors. Serine proteases, particularly NE, degrade cytoskeletal elements, facilitating the occurrence of NETosis. In turn, calprotectin is released by neutrophils in inflammation, serving as an alarmin with pro-inflammatory functions and contributing to the anti-microbial factors within NETs. Its importance in infection clearance has been acknowledged [42].

Previously, it was demonstrated that distinct signals originating from both bacteria and activated platelets can trigger the process of NETosis. This phenomenon was explored by Chiau-Jing Jung and Chiou-Yueh Yeh in 2015 [43]. The authors proposed a mechanism in which IE pathogens form aggregates with platelets on heart valves, stimulating platelets through IgG molecules bearing the Fcγ receptor. This activation process leads to the expression of P-selectin, a component found in platelet α-granules and becomes expressed only upon activation. The increase in P-selectin expression is succeeded by the creation of platelet–leukocyte conjugates through the attachment to leukocytes, facilitated by the P-selectin Glycoprotein Ligand 1 receptor [44,45]. The results of this study confirmed the upregulation of P-selectin in vegetations compared to the cardiac leaflets. Furthermore, a higher P-selectin expression was found in non-*Bartonella* spp.-caused IE cases; however, the difference was not statistically significant. The activation of platelets involves several signaling pathways, including Src family kinases, Syk, PI3K, and p38 MAPK [46]. Furthermore, bacteria also play a role in this process by inducing reactive oxygen species production and histone citrullination in neutrophils. This activation is facilitated through interactions with the Fcγ receptor and TLR2. The culmination of these events results in the creation of NETs. These NETs serve a dual purpose: they contribute to the activation of coagulation, platelet aggregation, fibrin formation, and deposition. These multifaceted effects of NETs on the coagulation system highlight their significant role in elevating the risk of thrombosis [47]. This entrapment mechanism aids in the expansion of vegetation during IE. Notably, recent studies have demonstrated that while the release of DNA to the extracellular milieu is primarily for the purpose of trapping and killing bacteria, it can also induce inflammation, which may lead to worsening disease pathology [48,49,50]. Importantly, beyond their involvement in IE, platelets and their associated P-selectin also play an active role in driving the progression of advanced atherosclerotic lesions. This fact has been discussed by other researchers [44,51]. Thus, in addition to the endothelium, platelets and their P-selectin also actively promote advanced atherosclerotic lesion development.

It has been shown that after activation, NE is released from azurophilic granules and moves to the nucleus, where it partially breaks down lamin and specific histones, facilitating nuclear chromatin decondensation. Later, MPO collaborates with NE to further promote chromatin decondensation [52]. Recent observations suggested the neutrophil proteases linked to NETs significantly affect the integrity of NET-associated proteins when inducing NET formation through the activation of isolated human neutrophils [53]. Furthermore, indications of the intricate nature of the immune response to polymicrobial infections were observed [54]. Understanding the role of the nucleus in NETosis offers valuable insights into the behavior of neutrophils in immune responses, especially in the context of inflammatory disorders. At the ultrastructural level, the decondensation of nuclear chromatin is a crucial stage that precedes nuclear breakdown in the process of NET release. This is observed through the swelling of the nuclei in neutrophils undergoing NETosis [55]. Several patterns of NETosis have been recognized to date [56]. Past TEM studies have distinctly shown the dissolution of the nucleus in both “suicidal” and “vital” NET release. In the case of “suicidal” NETosis, the nuclear envelope breaks down before decondensed chromatin fills the cytoplasm, blending with granule components, and resulting in the lytic release as a NET [57]. Earlier studies verified the detachment of the inner and outer nuclear membranes and the formation of vesicles containing nuclear DNA [58]. Notably, the initiation of NETosis may be linked to the inhibition of apoptosis, thereby amplifying the overall antimicrobial impact [59]. In this study, the application of transmission electron microscopy to analyze the ultrastructural characteristics of heart valve leaflets and vegetations in IE, particularly focusing on the role of neutrophilic leukocytes during NET formation, offered novel insights into cellular-level interactions and processes. We confirmed irregularities of the perinuclear space, sometimes filled with fine filamentous material, as well as ruptures of the nuclear envelope, with chromatin sandwiched between cytoplasmic organelles and components of the extracellular matrix. In turn, the use of scanning electron microscopy to visualize bacterial adhesion, fibrin network formation, and platelet appearance in vegetations in both *Bartonella* spp.-caused and non-*Bartonella* spp.-caused IE cases presented a visual depiction of the disease process at a microscale level. This approach was verified for its utilization in examining the structure of IE vegetations induced by various bacterial species, aiming to comprehend the associated pathophysiological mechanism of vegetation development [60,61].

NGS testing demonstrated its usefulness, especially in cases of BCNE, identifying the causative microorganism in 66.7% of cases and prompting changes in antibiotic therapy for 23.9% of patients. This highlights the clinical impact of NGS in guiding appropriate antimicrobial treatment when conventional methods fail to identify the causative pathogen. Comparative studies between BCNE and BCPE, as well as sepsis with known versus unknown causative agents, did not show significant outcome differences [33,62]. However, a recent major study from the EURO-ENDO registry confirmed higher long-term mortality rates in BCNE patients [63]. *Bartonella* spp. and *Coxiella burnetii* are the primary causative microorganisms in most BCNE cases [64]. Interestingly, this study found a higher than expected incidence of *Bartonella* spp. and no cases of *Coxiella* IE. Geographic location and local endemics may explain the differences observed compared to other studies in which *C. burnetii* was more common [65]. Previous studies have shown *Bartonella* spp. to be an emerging cause of BCNE [18]. Homelessness and alcoholism were mentioned as the significant risk factors for developing *Bartonella* spp. IE in the study conducted in France and published by Raoult et al. [66]. This finding is in line with our findings, where alcoholism history was observed significantly more often in *Bartonella* spp. IE than for other microorganism-caused IE. Infections caused by *Bartonella* spp. are typically insect vector-borne, where cat flea, human body louse, and sand fly are the most frequent transmitters [67]. The prevalence of alcoholism as well as body lice and other ectoparasites in homeless persons are high, so this is the most likely explanation for high *Bartonella* spp. infections and IE in this group of patients [68]. Treatment with aminoglycoside antibiotics showed better outcomes in *Bartonella* spp. IE cases [66]. Moreover, *Bartonella* spp. has been associated with extracardiac manifestations, particularly renal involvement. In the study by Ehrlich et al., 44% of patients with *Bartonella* spp. IE evidenced renal involvement, including glomerulonephritis and acute kidney injury [69,70]. The presence of higher creatinine levels observed in *Bartonella*-caused IE patients compared to other microorganisms was also confirmed in the given study.

The present study has some limitations to consider when interpreting the findings regarding the 16S rRNA NGS method. Firstly, it was conducted at a single center with a relatively small sample size, potentially limiting generalizability. Patient selection was also not comprehensive, as only randomly selected cases were included, affecting the detected microorganism proportions. Secondly, the detection of *Bartonella* spp. was limited to the genus level, not allowing the identification of specific species, which could have provided additional insights. Simultaneously, the use of 16S rRNA NGS for detecting *Bartonella* spp. has promising aspects; however, it comes with drawbacks, including variable sensitivity, lack of processing consensus, and inability to differentiate viable from non-viable microorganisms. Additionally, 16S rRNA NGS cannot detect fungi and viruses, which can cause IE in rare cases, as was demonstrated in the study by Badiee [71]. Thirdly, the presence of contamination is a potential concern in the study. Although many samples showed an admixture of other microorganism DNA, in most cases, one microorganism had significantly higher relative abundance (above 90%). Notably, in eight samples of *Bartonella* spp., the relative abundance was 100%, and in two samples, it ranged from 95.4% to 99.6%, strongly indicating *Bartonella* spp. as the causative agent. In contrast, other microorganism-caused IE cases had a relative abundance of 100% in only 10.9% of samples. The authors hypothesize that inappropriate antibiotic therapy, not targeting *Bartonella* spp., may have influenced these findings. However, no deeper analysis regarding antibiotic treatment was done, which is another drawback of this study. Additionally, this study involved the examination of vegetation from both mitral valves and aortic valves. This factor could potentially contribute to differential effects on vegetation formation. Moreover, while this study delved into the intricacies of NET formation in cases of *Bartonella* spp.-caused and non-*Bartonella* spp.-caused IE, it did not aim to provide a step-by-step analysis of NET formation. Instead, its focus remained on observing the occurrence of this process within the collected samples at the time of surgery.

Collectively, BCNE poses a diagnostic challenge, necessitating a multidisciplinary approach and a high level of suspicion. Individualized diagnostic testing and management are crucial, considering the underlying pathogen, clinical severity, and comorbidities. Clinicians should be mindful of fastidious microorganisms, prior antibiotic use, and low bacterial inoculum as potential causes of negative blood cultures in suspected endocarditis cases. The study advocates for the use of NGS on excised heart valves as an effective tool for diagnosing IE, particularly when blood cultures yield negative results. Further research is needed to establish the clinical utility of NGS in routine practice and validate the association between blood culture negativity and worse outcomes in IE patients. The study reveals significant differences between *Bartonella* spp.-caused and non-*Bartonella* spp.-caused IE cases in terms of patients’ characteristics, laboratory indices, and clinical outcomes. This nuanced understanding of how different pathogens affect patients’ health contributes to a more comprehensive understanding of the disease.

## 5. Conclusions

Patients with IE caused by *Bartonella* spp. often exhibit a background of alcoholism, elevated levels of creatinine and BNP, and lower ejection fraction of the left ventricle, lower leukocyte, platelet, and glucose levels compared to individuals with non-*Bartonella* spp. IE. The activation markers of neutrophils in valvular leaflets were more prominently observed in non*-Bartonella* spp. IE patients than in those with *Bartonella* spp. IE. The application of 16S rRNA NGS proves to be a valuable diagnostic tool for identifying microorganisms that are challenging to diagnose using standard microbiological methods.

## Figures and Tables

**Table 1:** The primers used in this study with Illumina overhang adapters to amplify the 16S rRNA V3-4 region.

Name	Sequence (Illumina Adapter, Heterogeneity Spacer, 16S Region Primer)	Reference
16S V3 Fw (341F)	TCGTCGGCAGCGTCAGATGTGTATAAGAGACAGNNNNNNCCTACGGGNGGCWGCAG	[27]
16S V4 Rs (805R)	GTCTCGTGGGCTCGGAGATGTGTATAAGAGACAGNNNNNNGACTACHVGGGTATCTAATCC	[27]

**Table 2:** Comparison of non-*Bartonella* spp.- and *Bartonella* spp.-caused IE patients’ characteristics, laboratory indices, and clinical outcomes.

Characteristic	Non-*Bartonella* spp. IE, *N* = 35	*Bartonella* spp. IE, *N* = 11	*p* Value
Age, years, mean (SD)	55.6 (14.8)	54.6 (9.8)	*p* = 0.8217
Sex, male, N (%)	26 (74.3)	10 (9.9)	*p*_Y_ = 0.4551
EuroScore II risk score, %, median (IQR)	6.5 (3.6–14.3)	6.3 (3.6–12.6)	*p*_MW_ = 0.9442
IE mortality risk score, %, median (IQR)	30.2 (13.6–41.1)	30.2 (21.5–34.9)	*p*_MW_ = 0.7988
Charlson Comorbidity index, points, median (IQR)	2.5 (1–5)	4 (3–5)	*p*_MW_ = 0.1868
Diabetes mellitus, N (%)	8 (22.9)	2 (18.2)	*p*_Y_ = 0.9274
Alcoholism history, N (%)	5 (14.3)	7 (63.6)	***p*** **_Y_** ** = 0.0124**
Intravenous drug usage history, N (%)	3 (8.6)	0 (0.0)	*p*_Y_ = 0.7609
Length of vegetation, mm, median (IQR)	12.0 (0.0–18.0)	13.0 (12.0–22.0)	*p*_MW_ = 0.1644
Embolism, N (%)	8 (22.9)	3 (27.3)	*p*_Y_ = 0.9158
Cerebral embolism, N (%)	7 (20.0)	2 (18.2)	*p*_Y_ = 0.7618
Spleen embolism, N (%)	1 (2.9)	1 (9.1)	*p*_Y_ = 0.9706
Kidney embolism, N (%)	2 (5.7)	0 (0.0)	*p*_Y_ = 0.9706
EF of the left ventricle, %, mean (SD)	56.4 (7.8)	50.6 (9.4)	***p*** ** = 0.0481**
Right ventricle systolic pressure, mm/Hg, median (IQR)	37.5 (30.0–56.3)	46.5 (43.8–56.3)	*p*_MW_ = 0.0627
Leukocytes, count 10^9^ mL, median (IQR)	7.6 (5.8–9.7)	5.2 (4.2–6.0)	***p*** **_MW_** ** = 0.0038**
Platelets, count 10^9^ mL, median (IQR)	241.0 (186.0–325.0)	174.0 (82.0–218.0)	***p*** **_MW_** ** = 0.0185**
Red blood cells, count 10^9^ mL, median (IQR)	3.7 (3.2–4.4)	3.6 (3.1–4.1)	*p*_MW_ = 0.1987
Hemoglobin, g/L, mean (SD)	110.4 (22.2)	99.1 (14.1)	*p* = 0.1205
CRP level before surgery, mg/dL, median (IQR)	32.8 (7.5–55.1)	33.5 (6.6–57.0)	*p*_MW_ = 0.7837
CRP level 2nd day after surgery, mg/dL, median (IQR)	185.8 (133.9–228.8)	157.8 (94.2–220.6)	*p*_MW_ = 0.2979
CRP level 4th day after surgery, mg/dL, median (IQR)	110.0 (72.7–159.3)	118.7 (104.8–215.1)	*p*_MW_ = 0.4110
CRP level 6th day after surgery, mg/dL, median (IQR)	57.6 (31.0–100.5)	71.9 (46.3–101.3)	*p*_MW_ = 0.4572
Procalcitonin, ng/mL, median (IQR)	0.12 (0.08–0.28)	0.22 (0.07–0.38)	*p*_MW_ = 0.4911
BNP, ng/mL, median (IQR)	429.8 (136.4–960.6)	1523.0 (692.9–3708.0)	***p*** **_MW_** ** = 0.0002**
Glucose, mkmol/L, median (IQR)	5.4 (4.8–6.7)	4.7 (4.3–5.6)	***p*** **_MW_** ** = 0.0375**
Creatinine, mmol/L, median (IQR)	77.0 (64.0–89.0)	104.0 (78.0–283.0)	***p*** **_MW_** ** = 0.0071**
Blood loss, first day, mL, median (IQR)	372.5 (250.0–630.0)	420.0 (301.3–930.0)	*p*_MW_ = 0.3463
Resternotomy due to acute bleeding, N (%)	6 (17.1)	3 (27.3)	*p*_Y_ = 0.7618
Mechanical lung ventilation, hours, median (IQR)	11.5 (8.0–72.0)	14.0 (9.0–25.0)	*p*_MW_ = 0.7533
Usage of vasopressors, N (%)	23 (65.7)	8 (72.7)	*p*_Y_ = 0.9489
Usage of beta-agonists, N (%)	13 (37.1)	7 (63.6)	*p*_Y_ = 0.2311
Days intrahospital, median (IQR)	31.0 (25.0–48.0)	37.0 (19.0–44.0)	*p*_MW_ = 0.5544
Days in the ICU, median (IQR)	3.0 (1.8–9.0)	3.0 (2.0–8.0)	*p*_MW_ = 0.9106
Intrahospital death, N (%)	4 (11.4)	0 (0.0)	*p*_Y_ = 0.5755

Abbreviations: IE—infective endocarditis, SD—standard deviation, IQR—interquartile range, EF—ejection fraction, CRP—C-reactive protein, BNP—Beta-type natriuretic peptide, ICU—intensive care unit, *p*—*p*-values from unpaired *t*-test, *p*_MW_—*p*-values from Mann–Whitney U-test, *p*_Y_—*p*-values from Chi_2_ test with Yates correction.

## Data Availability

Data are available from the corresponding author upon reasonable request, preferentially by e-mail: kristians.meidrops@stradini.lv.

## References

[1] Talha K.M., Baddour L.M., Thornhill M.H., Arshad V., Tariq W., Tleyjeh I.M., Scott C.G., Hyun M.H., Bailey K.R., Anavekar N.S. (2021). Escalating incidence of infective endocarditis in Europe in the 21st century. Open Hear..

[2] Lisi M., Flamigni F., Russo M., Cameli M., Mandoli G.E., Pastore M.C., Mele D., Campo G., Henein M.Y., Rubolli A. (2023). Incidence and mortality of infective endocarditis in the last decade: A single center study. J. Cardiovasc. Med..

[3] Rajani R., Klein J.L. (2020). Infective endocarditis: A contemporary update. Clin. Med..

[4] Sexton D.J., Spelman D. (2003). Current best practices and guidelines. Assessment and management of complications in infective endocarditis. Cardiol. Clin..

[5] Chirillo F. (2021). New approach to managing infective endocarditis. Trends Cardiovasc. Med..

[6] Salem M., Friedrich C., Saad M., Frank D., Salem M., Puehler T., Schoettler J., Schoeneich F., Cremer J., Haneya A. (2021). Active Infective Native and Prosthetic Valve Endocarditis: Short- and Long-Term Outcomes of Patients after Surgical Treatment. J. Clin. Med..

[7] Luaces M., Vilacosta I., Fernández C., Sarriá C., San Román J.A., Graupner C., Núñez-Gil I.J. (2009). Vegetation size at diagnosis in infective endocarditis: Influencing factors and prognostic implications. Int. J. Cardiol..

[8] Leitman M., Dreznik Y., Tyomkin V., Fuchs T., Krakover R., Vered Z. (2012). Vegetation size in patients with infective endocarditis. Eur. Heart J. Cardiovasc. Imaging.

[9] Vikram H.R. (2007). The long and short of vegetations in infective endocarditis. Expert Rev. Anti-Infect. Ther..

[10] Kamde S.P., Anjankar A. (2022). Pathogenesis, Diagnosis, Antimicrobial Therapy, and Management of Infective Endocarditis, and Its Complications. Cureus.

[11] Braï M.A., Hannachi N., El Gueddari N., Baudoin J.P., Dahmani A., Lepidi H., Habib G., Camoin-Jau L. (2023). The Role of Platelets in Infective Endocarditis. Int. J. Mol. Sci..

[12] Luehr M., Bauernschmitt N., Peterss S., Li Y., Heyn O., Dashkevich A., Oberbach A., Bagaev E., Pichlmaier M.A., Juchem G. (2020). Incidence and Surgical Outcomes of Patients with Native and Prosthetic Aortic Valve Endocarditis. Ann. Thorac. Surg..

[13] Meyers S., Crescente M., Verhamme P., Martinod K. (2022). *Staphylococcus aureus* and Neutrophil Extracellular Traps: The Master Manipulator Meets Its Match in Immunothrombosis. Arter. Thromb. Vasc. Biol..

[14] Nappi F., Martuscelli G., Bellomo F., Singh S.S.A., Moon M.R. (2022). Infective Endocarditis in High-Income Countries. Metabolites.

[15] Haddad S.F., DeSimone D.C., Chesdachai S., Gerberi D.J., Baddour L.M. (2022). Utility of Metagenomic Next-Generation Sequencing in Infective Endocarditis: A Systematic Review. Antibiotics.

[16] Suardi L.R., de Alarcon A., Garcia M.V., Ciezar A.P., Hidalgo Tenorio C., Martinez-Marcos F.J., Concejo-Martinez E., De la Torre Lima J., Vinuesa Garcia D., Luque Marquez R. (2021). Blood culture-negative infective endocarditis: A worse outcome? Results from a large multicentre retrospective Spanish cohort study. Infect. Dis..

[17] Scheer C.S., Fuchs C., Gründling M., Vollmer M., Bast J., Bohnert J.A., Zimmermann K., Hahnenkamp K., Rehberg S., Kuhn S.O. (2019). Impact of antibiotic administration on blood culture positivity at the beginning of sepsis: A prospective clinical cohort study. Clin. Microbiol. Infect..

[18] Okaro U., Addisu A., Casanas B., Anderson B. (2017). Bartonella Species, an Emerging Cause of Blood-Culture-Negative Endocarditis. Clin. Microbiol. Rev..

[19] Liesenborghs L., Meyers S., Vanassche T., Verhamme P. (2020). Coagulation: At the heart of infective endocarditis. J. Thromb. Haemost..

[20] Hidalgo A., Libby P., Soehnlein O., Aramburu I.V., Papayannopoulos V., Silvestre-Roig C. (2022). Neutrophil extracellular traps: From physiology to pathology. Cardiovasc. Res..

[21] Tanaka Y., Yamanaka N., Koyano I., Hasunuma I., Kobayashi T., Kikuyama S., Iwamuro S. (2022). Dual Roles of Extracellular Histone H3 in Host Defense: Its Differential Regions Responsible for Antimicrobial and Cytotoxic Properties and Their Modes of Action. Antibiotics.

[22] Meyers S., Lox M., Kraisin S., Liesenborghs L., Martens C.P., Frederix L., Van Bruggen S., Crescente M., Missiakas D., Baatsen P. (2023). Neutrophils Protect Against *Staphylococcus aureus* Endocarditis Progression Independent of Extracellular Trap Release. Arter. Thromb. Vasc. Biol..

[23] Martin D.R., Witten J.C., Tan C.D., Rodriguez E.R., Blackstone E.H., Pettersson G.B., Seifert D.E., Willard B.B., Apte S.S. (2020). Proteomics identifies a convergent innate response to infective endocarditis and extensive proteolysis in vegetation components. J. Clin. Investig..

[24] Huang L., Lu W., Ning Y., Liu J. (2022). Reverse effects of Streptococcus mutans physiological states on neutrophil extracellular traps formation as a strategy to escape neutrophil killing. Front. Cell. Infect. Microbiol..

[25] Kaplan M.J., Radic M. (2012). Neutrophil Extracellular Traps: Double-Edged Swords of Innate Immunity. J. Immunol..

[26] Jenne C.N., Wong C.H., Zemp F.J., McDonald B., Rahman M.M., Forsyth P.A., McFadden G., Kubes P. (2013). Neutrophils Recruited to Sites of Infection Protect from Virus Challenge by Releasing Neutrophil Extracellular Traps. Cell Host Microbe.

[27] Fadrosh D.W., Ma B., Gajer P., Sengamalay N., Ott S., Brotman R.M., Ravel J., Fadrosh D.W., Ma B., Gajer P. (2014). An improved dual-indexing approach for multiplexed 16S rRNA gene sequencing on the Illumina MiSeq platform. Microbiome.

[28] Bolger A.M., Lohse M., Usadel B. (2014). Trimmomatic: A flexible trimmer for Illumina sequence data. Bioinformatics.

[29] Callahan B.J., Mcmurdie P.J., Rosen M.J., Han A.W., Johnson A.J.A., Holmes S.P. (2016). DADA_2_: High-resolution sample inference from Illumina amplicon data. Nat. Methods.

[30] Rognes T., Flouri T., Nichols B., Quince C., Mahé F. (2016). VSEARCH: A versatile open source tool for metagenomics. PeerJ.

[31] Katoh K., Standley D.M. (2013). MAFFT Multiple Sequence Alignment Software Version 7: Improvements in Performance and Usability. Mol. Biol. Evol..

[32] Price M.N., Dehal P.S., Arkin A.P. (2010). FastTree 2—Approximately Maximum-Likelihood Trees for Large Alignments. PLoS ONE.

[33] Pedregosa F. (2011). Scikit-learn: Machine learning in Python. J. Mach. Learn. Res..

[34] Garvey W. (1996). Silver Impregnation Techniques to Identify Spirochetes and Other Bacteria. J. Histotechnol..

[35] McCormick J.K., Tripp T.J., Dunny G.M., Schlievert P.M. (2002). Formation of Vegetations during Infective Endocarditis Excludes Binding of Bacterial-Specific Host Antibodies to *Enterococcus faecalis*. J. Infect. Dis..

[36] Chavakis T., Bierhaus A., Al-Fakhri N., Schneider D., Witte S., Linn T., Nagashima M., Morser J., Arnold B., Preissner K.T. (2003). The pattern recognition receptor (rage) is a counterreceptor for leukocyte integrins: A novel pathway for inflammatory cell recruitment. J. Exp. Med..

[37] Shaw S.K., Ma S., Kim M.B., Rao R.M., Hartman C.U., Froio R.M., Yang L., Jones T., Liu Y., Nusrat A. (2004). Coordinated Redistribution of Leukocyte LFA-1 and Endothelial Cell ICAM-1 Accompany Neutrophil Transmigration. J. Exp. Med..

[38] Chavakis T., Preissner K.T., Herrmann M. (2007). The anti-inflammatory activities of Staphylococcus aureus. Trends Immunol..

[39] Thiene G., Basso C. (2006). Pathology and pathogenesis of infective endocarditis in native heart valves. Cardiovasc. Pathol..

[40] Jensen H.E., Gyllensten J., Hofman C., Leifsson P.S., Agerholm J.S., Boye M., Aalbæk B. (2010). Histologic and Bacteriologic Findings in Valvular Endocarditis of Slaughter-Age Pigs. J. Veter- Diagn. Investig..

[41] Patel S., Richert M.E., White R., Lambing T., Saleeb P. (2019). A Case of Bartonella Quintana Culture-Negative Endocarditis. Am. J. Case Rep..

[42] Urban C.F., Ermert D., Schmid M., Abu-Abed U., Goosmann C., Nacken W., Brinkmann V., Jungblut P.R., Zychlinsky A. (2009). Neutrophil Extracellular Traps Contain Calprotectin, a Cytosolic Protein Complex Involved in Host Defense against Candida albicans. PLoS Pathog..

[43] Jung C.-J., Yeh C.-Y., Hsu R.-B., Lee C.-M., Shun C.-T., Chia J.-S. (2015). Endocarditis Pathogen Promotes Vegetation Formation by Inducing Intravascular Neutrophil Extracellular Traps Through Activated Platelets. Circ..

[44] LeGuyader A., Watanabe R., Berbé J., Boumediene A., Cogné M., Laskar M. (2006). Platelet activation after aortic prosthetic valve surgery. Interact. Cardiovasc. Thorac. Surg..

[45] Lam F.W., Rumbaut R.E. (2015). Platelets mediate acetaminophen hepatotoxicity. Blood.

[46] Pfister C., Pfrommer H., Tatagiba M.S., Roser F. (2012). Vascular endothelial growth factor signals through platelet-derived growth factor receptor β in meningiomas in vitro. Br. J. Cancer.

[47] Martos L., Oto J., Fernández-Pardo Á., Plana E., Solmoirago M.J., Cana F., Hervás D., Bonanad S., Ferrando F., España F. (2020). Increase of Neutrophil Activation Markers in Venous Thrombosis—Contribution of Circulating Activated Protein C. Int. J. Mol. Sci..

[48] Bhattacharya M., Berends E.T., Chan R., Schwab E., Roy S., Sen C.K., Torres V.J., Wozniak D.J. (2018). Staphylococcus aureus biofilms release leukocidins to elicit extracellular trap formation and evade neutrophil-mediated killing. Proc. Natl. Acad. Sci. USA.

[49] Bassani B., Cucchiara M., Butera A., Kayali O., Chiesa A., Palano M.T., Olmeo F., Gallazzi M., Dellavia C.P., Mortara L. (2023). Neutrophils&rsquo; Contribution to Periodontitis and Periodontitis-Associated Cardiovascular Diseases. Int. J. Mol. Sci..

[50] Koh C.C., Gollob K.J., Dutra W.O. (2023). Balancing the functions of DNA extracellular traps in intracellular parasite infections: Implications for host defense, disease pathology and therapy. Cell Death Dis..

[51] Burger P.C., Wagner D.D. (2003). Platelet P-selectin facilitates atherosclerotic lesion development. Blood.

[52] Papayannopoulos V., Metzler K.D., Hakkim A., Zychlinsky A. (2010). Neutrophil elastase and myeloperoxidase regulate the formation of neutrophil extracellular traps. J. Cell Biol..

[53] de Bont C., Pruijn G.J.M. (2023). Citrulline is not a major determinant of autoantibody reactivity to neutrophil extracellular traps. Philos. Trans. R. Soc. B Biol. Sci..

[54] Kao P.H.-N., Ch’Ng J.-H., Chong K.K.L., Stocks C.J., Wong S.L., A Kline K. (2023). *Enterococcus faecalis* suppresses *Staphylococcus aureus*-induced NETosis and promotes bacterial survival in polymicrobial infections. FEMS Microbes.

[55] Neubert E., Meyer D., Rocca F., Günay G., Kwaczala-Tessmann A., Grandke J., Senger-Sander S., Geisler C., Egner A., Schön M.P. (2018). Chromatin swelling drives neutrophil extracellular trap release. Nat. Commun..

[56] Delgado-Rizo V., Martínez-Guzmán M.A., Iñiguez-Gutierrez L., García-Orozco A., Alvarado-Navarro A., Fafutis-Morris M. (2017). Neutrophil Extracellular Traps and Its Implications in Inflammation: An Overview. Front. Immunol..

[57] Manley H.R., Keightley M.C., Lieschke G.J. (2018). The Neutrophil Nucleus: An Important Influence on Neutrophil Migration and Function. Front. Immunol..

[58] Pilsczek F.H., Salina D., Poon K.K.H., Fahey C., Yipp B.G., Sibley C.D., Robbins S.M., Green F.H.Y., Surette M.G., Sugai M. (2010). A Novel Mechanism of Rapid Nuclear Neutrophil Extracellular Trap Formation in Response to *Staphylococcus aureus*. J. Immunol..

[59] Vorobjeva N.V., Chernyak B.V. (2020). NETosis: Molecular Mechanisms, Role in Physiology and Pathology. Biochemistry.

[60] Onouchi T., Shiogama K., Mizutani Y., Takaki T., Tsutsumi Y. (2016). Visualization of Neutrophil Extracellular Traps and Fibrin Meshwork in Human Fibrinopurulent Inflammatory Lesions: III. Correlative Light and Electron Microscopic Study. Acta Histochem. ET Cytochem..

[61] Hannachi N., Lepidi H., Fontanini A., Takakura T., Bou-Khalil J., Gouriet F., Habib G., Raoult D., Camoin-Jau L., Baudoin J.-P. (2020). A Novel Approach for Detecting Unique Variations among Infectious Bacterial Species in Endocarditic Cardiac Valve Vegetation. Cells.

[62] Zamorano J., Sanz J., Moreno R., Almerıa C., Rodrigo J.-L., Samedi M., Herrera D., Aubele A., Mataix L., Serra V. (2001). Comparison of outcome in patients with culture-negative versus culture-positive active infective endocarditis. Am. J. Cardiol..

[63] Kong W.K., Salsano A., Giacobbe D.R., A Popescu B., Laroche C., Duval X., Schueler R., Moreo A., Colonna P., Piper C. (2022). Outcomes of culture-negative vs. culture-positive infective endocarditis: The ESC-EORP EURO-ENDO registry. Eur. Hear. J..

[64] Godfrey R., Curtis S., Schilling W.H., James P.R. (2020). Blood culture negative endocarditis in the modern era of 16S rRNA sequencing. Clin. Med..

[65] Wang W., Chen O., Liu W., Gan L., Li X., Ma Q., Hu X., Jian X. (2022). *Coxiella burnetii* and *Bartonella* Endocarditis Diagnosed by Metagenomic Next-Generation Sequencing. J. Clin. Med..

[66] Raoult D., Fournier P.-E., Vandenesch F., Mainardi J.-L., Eykyn S.J., Nash J., James E., Benoit-Lemercier C., Marrie T.J. (2003). Outcome and Treatment of Bartonella Endocarditis. Arch. Intern. Med..

[67] Breitschwerdt E.B. (2017). Bartonellosis, One Health and all creatures great and small. Veter. Dermatol..

[68] Bonilla D.L., Cole-Porse C., Kjemtrup A., Osikowicz L., Kosoy M. (2014). Risk Factors for Human Lice and Bartonellosis among the Homeless, San Francisco, California, USA. Emerg. Infect. Dis..

[69] Ehrlich G.D., Ahmed A., Earl J., Hiller N.L., Costerton J.W., Stoodley P., Post J.C., DeMeo P., Hu F.Z. (2010). The distributed genome hypothesis as a rubric for understanding evolution in situ during chronic bacterial biofilm infectious processes. FEMS Immunol. Med Microbiol..

[70] Raybould J.E., Raybould A.L., Morales M.K., Zaheer M., Lipkowitz M.S., Timpone J.G., Kumar P.N. (2016). Bartonella Endocarditis and Pauci-Immune Glomerulonephritis: A Case Report and Review of the Literature. Infect. Dis. Clin. Pract..

[71] Badiee P., Amirghofran A.A., Nour M.G., Shafa M., Nemati M.H. (2014). Incidence and Outcome of Documented Fungal Endocarditis. Int. Cardiovasc. Res. J..

